# Investigating Ligand Sphere Perturbations on Mn^III^–Alkylperoxo Complexes

**DOI:** 10.3390/molecules29081849

**Published:** 2024-04-18

**Authors:** Samuel A. Brunclik, Elizabeth N. Grotemeyer, Zahra Aghaei, Mohammad Rasel Mian, Timothy A. Jackson

**Affiliations:** 1 Department of Chemistry and Center for Environmentally Beneficial Catalysis, University of Kansas, Lawrence, KS 66045, USA; bruncliksam@ku.edu (S.A.B.); egrotemeyer1@gmail.com (E.N.G.); zahra.aghaei@ku.edu (Z.A.); 2 Protein Structure and X-ray Crystallography Laboratory, University of Kansas, Lawrence, KS 66045, USA; rasel@ku.edu

**Keywords:** alkylperoxo, manganese catalysts, O–O activation, ligand sphere, substrate oxidation

## Abstract

Manganese catalysts that activate hydrogen peroxide carry out several different hydrocarbon oxidation reactions with high stereoselectivity. The commonly proposed mechanism for these reactions involves a key manganese(III)-hydroperoxo intermediate, which decays via O–O bond heterolysis to generate a Mn(V)–oxo species that institutes substrate oxidation. Due to the scarcity of characterized Mn^III^–hydroperoxo complexes, Mn^III^–alkylperoxo complexes are employed to understand factors that affect the mechanism of the O–O cleavage. Herein, we report a series of novel complexes, including two room-temperature-stable Mn^III^–alkylperoxo species, supported by a new amide-containing pentadentate ligand (^6Me^dpaq^5NO2^). We use a combination of spectroscopic methods and density functional theory computations to probe the effects of the electronic changes in the ligand sphere *trans* to the hydroxo and alkylperoxo units to thermal stability and reactivity. The structural characterizations for both Mn^II^(OTf)(^6Me^dpaq^5NO2^) and [Mn^III^(OH)(^6Me^dpaq^5NO2^)](OTf) were obtained via single-crystal X-ray crystallography. A perturbation to the ligand sphere allowed for a marked increase in reactivity towards an organic substrate, a modest change in the distribution of the O–O cleavage products from homolytic and heterolytic pathways, and little change in thermal stability.

## 1. Introduction

Manganese-dependent enzymes are vital to a variety of organisms due to their role in carrying out important oxidation-reduction reactions [1,2,3,4,5,6,7,8,9,10,11,12,13]. For example, manganese superoxide dismutase (MnSOD) and manganese lipoxygenase (MnLOX) detoxify free radicals and convert polyunsaturated fatty acids into alkyl hydroperoxides, respectively [1,2,3,4,14,15,16,17]. Inspired by these and other Mn enzymes, a range of synthetic manganese catalysts have been developed to carry out a variety of highly stereo-selective oxidation reactions [18], including asymmetric olefin epoxidation and late-stage C–H bond functionalization [19,20,21,22,23,24,25,26,27]. Due to the abundance and low cost of manganese, as well as the low environmental impact of obtaining manganese, increasing the use of such synthetic catalysts in the industry could reduce the environmental impacts of the current chemical processes [28,29].

At present, the proposed catalytic mechanisms for these synthetic manganese catalysts invoke oxygen–oxygen heterolysis of a Mn^III^–hydroperoxo intermediate to generate a manganese(V)–oxo species as the active oxidant [27,30]. However, due to the scarcity of Mn^III^–hydroperoxo complexes that have been trapped and spectroscopically characterized, there is a key gap in our understanding of the mechanism of this O–O cleavage step [31,32,33,34,35]. Nam and co-workers have previously reported Mn^III^–hydroperoxo complexes supported by tetramethylcyclam (TMC) and a ring-contracted derivative [33,34]. These complexes have shown intense electronic absorption bands in the UV region from ~300–400 nm. Resonance Raman spectra of the [Mn^III^(OOH)(TMC)]^2+^ complex revealed a band at 792 cm^−1^ that was sensitive to ^16^O/^18^O labeling [34]. Both of these complexes were shown to oxidize thioanisole, but only one could oxidize hydrocarbons [33,34]. However, the studies of the reactivity of these manganese(III)–hydroperoxo complexes primarily focused on their ability to react directly with various substrates and not on the pathways for O–O activation [33,34]. 

**Figure 1:**
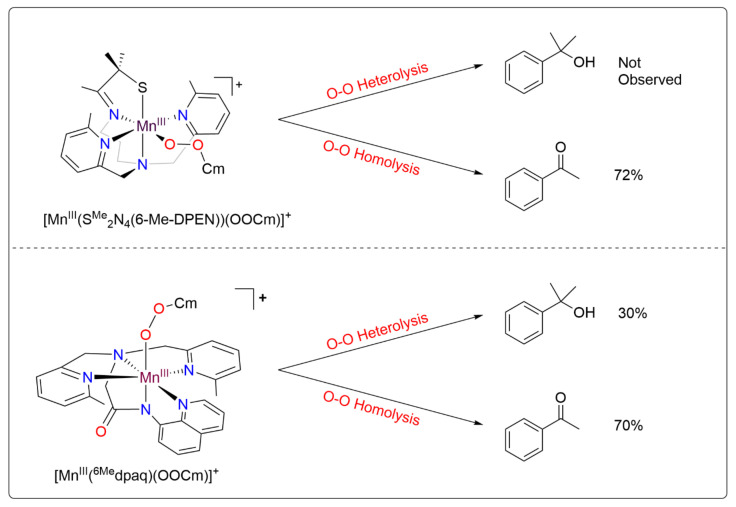
Product distribution of the cumylperoxo ligand following the thermal decay of [Mn^III^(OOCm)(N4S)]^+^ and [Mn^III^(OOCm)(^6Me^dpaq)]^+^.

Metal–alkylperoxo complexes, which are typically more stable than their metal–hydroperoxo analogs, have often been used to investigate the factors that affect O–O cleavage pathways [36,37,38,39,40,41]. For manganese, this use of alkylperoxo complexes has been justified by a similar performance by some Mn catalysts using either hydrogen peroxide (H_2_O_2_) or cumene hydroperoxide (CmOOH) as an oxidant, suggesting a common O–O activation mechanism between the two [42,43]. Previous studies of Mn^III^–alkylperoxo complexes supported by pentadentate N4S ligands by Kovacs and co-workers have yielded unique structure-reactivity correlations (the N4S ligand denotes a ligand with four nitrogen donors and one thiolate donor) [44]. For example, complexes with shorter O–O bonds yielded slower thermal decay rates. In addition, the O–O bond lengths were shown to be inversely related to the Mn–N bond lengths of the equatorial 6-Me-pyridyl or quinolyl ligands (see Figure 1 top for a representative structure). Further study of the Mn^III^–cumylperoxo complexes in this series revealed an exclusive formation of acetophenone as the organic product, which suggests O–O homolysis (as a heterolytic cleavage would produce 2-phenyl-2-propanol; see Figure 1) [44,45]. Taken together, the observations for this series of complexes establish that the local geometry around Mn^III^alkyl–peroxo centers controls the rate of decay for O–O homolysis. However, these findings stand in contrast to the proposed heterolytic mechanism for the Mn^III^–hydroperoxo intermediates formed in the reaction with H_2_O_2_. Recently, Kovacs and co-workers expanded this series of complexes by introducing an alkoxide-containing derivative [46], [Mn^III^(OO*^t^*Bu)(N4O)]^+^ (the N4O ligand denotes a ligand with four nitrogen donors and one alkoxide donor). The markedly greater thermal stability of this complex further underscored the relationship between the primary coordination sphere and thermal decay rates.

In our effort to improve our understanding of the role of the ligand environment in controlling the properties of Mn^III^–alkylperoxo complexes, we produced a series of Mn^III^–OOR complexes supported by amide-containing N5 ligands (see Figure 1 for a representative example) [47]. The initial complexes in this series were only thermally stable at lower temperatures (−15 °C), and a large excess of *^t^*BuOOH was required to form the Mn^III^–alkylperoxo complexes in high yield [47]. Seeking to improve the thermal stability of these complexes, we followed the work of Kovacs and co-workers and introduced 6-Me-pyridyl donors that enforced longer equatorial Mn–N bonds [44]. Two new complexes were generated using these ligand derivatives, and the increased thermal stability of these complexes at 25 °C allowed us to obtain the X-ray crystallographic structure of [Mn^III^(OOCm)(^6Me^dpaq)](OTf) [48]. In contrast to that observed for the N4S complexes, these complexes had high thermal stability but showed direct reactivity with triphenylphosphine to yield triphenylphosphine oxide and Mn^II^ products [48]. 

Further investigation into the geometric and electronic differences of these Mn^III^–OOR complexes, using density functional theory (DFT) compared with the experimental results, showed that the [Mn^III^(OO*^t^*Bu)(N4S)]^+^ complexes decay via a two-state mechanism for the O–O homolysis that is favored by the presence of π-acidic sulfur [49]. This two-state mechanism involves a crossing from the quintet spin state to the triplet spin state as the bond cleavage progresses [49]. In contrast, the [Mn^III^(OO*^t^*Bu)(^6Me^dpaq)]^+^ complexes follow a single-state reaction pathway for homolysis, remaining in the quintet spin state [49]. The lack of a lower-lying triplet pathway for O–O homolysis for [Mn^III^(OO*^t^*Bu)(^6Me^dpaq)]^+^ was consistent with the high thermal stability of this complex. Experimental analysis of the thermal decay of [Mn^III^(OOCm)(^6Me^dpaq)]^+^ in several solvents revealed acetophenone as a major product, supporting O–O homolysis as the dominant decay pathway (70% in benzonitrile) [48]. However, 2-phenyl-2-propanol was also observed as a decay product across all the solvents (30% in benzonitrile), showing that O–O heterolysis also occurs (Figure 1) [48]. This result was of particular interest because it differs from the exclusive decay of the analogous [Mn^III^(OOCm)(N4S)]^+^ complex by O–O homolysis and shows that changes to the ligand sphere can be used to favor O–O heterolysis [44,45]. 

On the basis of these results, we are interested in further probing the effect of the ligand sphere on the reactivity and decay pathways of Mn^III^–alkylperoxo complexes. Previous modifications of the ligand sphere of Mn^III^–OOR complexes had primarily been made to coordination sites *cis* to the alkylperoxo unit [48]. However, the site *trans* to the alkylperoxo unit is often very important when it comes to O–O cleavage (i.e., the so-called “push” effect) [50]. We, therefore, sought to develop a Mn^III^–alkylperoxo complex that would perturb the electronic properties *trans* to the alkylperoxo unit in order to further probe the effect of ligand sphere modifications on reactivity and decay pathways. 

**Figure 2:**
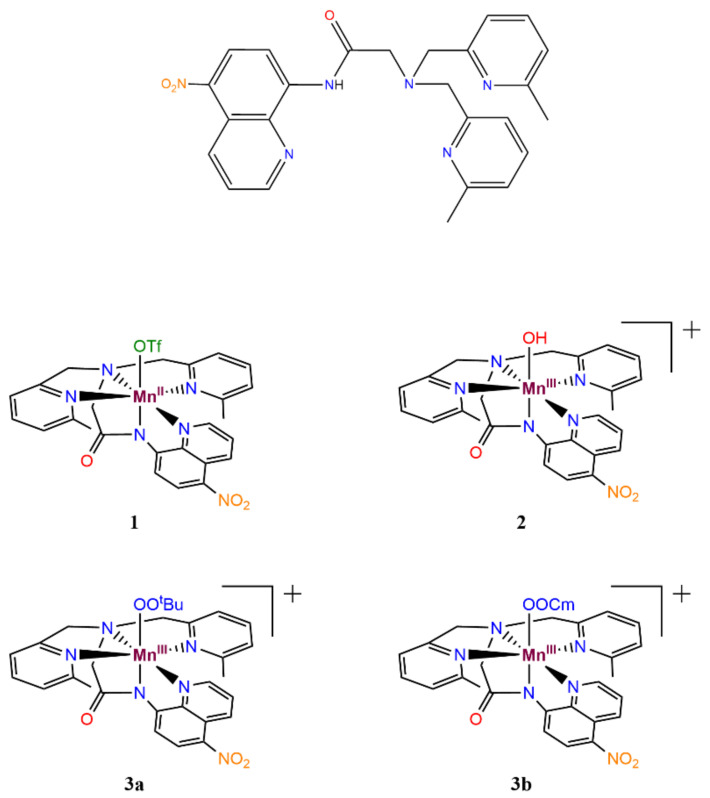
Molecular structures of H^6Me^dpaq^5NO2^ (2-(bis((6-methylpyridin-2-yl)methyl)amino)-*N-*(5-nitroquinolin-8-yl)acetamide), Mn^II^(OTf)(^6Me^dpaq^5NO2^) (**1**), [Mn^III^(OH)(^6Me^dpaq^5NO2^)](OTf) (**2**), [Mn^III^(OO*^t^*Bu)(^6Me^dpaq^5NO2^)](OTf) (**3a**), and [Mn^III^(OOCm)(^6Me^dpaq^5NO2^)](OTf) (**3b**).

Herein, we report Mn^II^, Mn^III^–hydroxo, and Mn^III^–alkylperoxo complexes, supported by a new ^6Me^dpaq^5NO2^ ligand (2-(bis((6-methylpyridin-2-yl)methyl)amino)-*N-*(5-nitroquinolin-8-yl)acetamide; see Figure 2). This new ligand incorporates a strongly electron-withdrawing nitro group at the 5 position of the quinoline, *para* to its connection with the axially bound amide group. We use a combination of experimental methods and DFT computations to probe the effects of the electronic changes in the ligand sphere *trans* to the hydroxo and alkylperoxo units on thermal stability and reactivity. The [Mn^III^(OH)(^6Me^dpaq^5NO2^)](OTf) (**2**) complex reacts stoichiometrically with either *^t^*BuOOH or CmOOH in CH_3_CN to form the [Mn^III^(OO*^t^*Bu)(^6Me^dpaq^5NO2^)]^+^ (**3a**) or [Mn^III^(OOCm)(^6Me^dpaq^5NO2^)]^+^ (**3b**) complexes, respectively. These Mn^III^–alkylperoxo complexes are stable in solution at room temperature with half-lives of ca. 4 days. The Mn^III^–alkylperoxo complexes follow the previously established trend for dpaq derivatives of high thermal stability while showing evidence for a direct reaction with phosphines in kinetic studies. These results further illuminate the key extent of control that the ligand sphere has over substrate reactivity and O–O cleavage pathways of Mn^III^–alkyperoxo adducts.

## 2. Results

### 2.1. Formation and Characterization of MnII(OTf)(^6Me^dpaq^5NO2^) *(****1****)*

**Figure 3:**
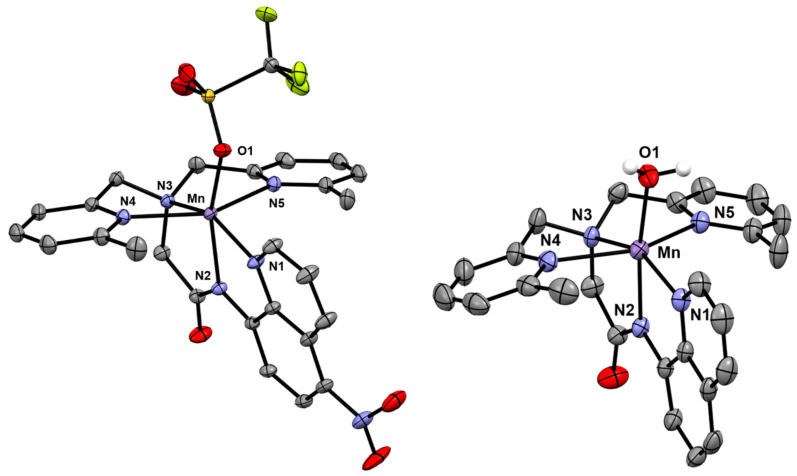
X-ray crystal structure of Mn^II^(OTf)(^6Me^dpaq^5NO2^) (**left**) and [Mn^II^(OH_2_)(^6Me^dpaq)](OTf) (**right**) [48]. Hydrogen atoms have been omitted for clarity. Ellipsoids, shown at 50% probability; disordered atoms in the nitro moiety omitted for clarity.

Complex **1** was prepared by the metallation of H^6Me^dpaq^5NO2^ with Mn^II^(OTf)_2_ in dichloromethane. The X-ray crystal structure of **1** reveals a monomeric, six-coordinate Mn^II^ center coordinated by the pentadentate ^6Me^dpaq^5NO2^ ligand and a triflate anion (Figure 3). The overall structure is similar to that of the previously reported [Mn^II^(OH_2_)(^6Me^dpaq)](OTf) complex [48], with the exception that a triflate ion completes that coordination sphere rather than a water molecule. When compared with [Mn^II^(OH_2_)(^6Me^dpaq)](OTf), the Mn–O1 bond of **1** is notably longer (2.108(3) vs. 2.169(7) Å, respectively; see Table 1), which likely reflects the weaker donating properties of the triflate anion. The Mn–N1 through Mn–N4 distances of **1** are all very similar to those of [Mn^II^(OH_2_)(^6Me^dpaq)](OTf), indicating that the electron-withdrawing property of the nitro group has little effect on the Mn^II^–N distances. In particular, the Mn–N(amido) distance (Mn–N2) of **1** is identical, within error, to that of [Mn^II^(OH_2_)(^6Me^dpaq)](OTf). The most notable Mn–N bond difference in **1** is the significant contraction of the Mn–N5 bond, as compared to that of [Mn^II^(H_2_O)(^6Me^dpaq)](OTf) (2.357(8) vs. 2.417(3) Å, respectively). This change brings the Mn–N4 and Mn–N5 bonds in **1** much closer in length to one another. 

The frozen-solution EPR spectrum of **1** at 9 K shows an intense six-line signal near *g* ≈ 2, consistent with a mononuclear S = 5/2 Mn^II^ species (Supplementary Materials Figure S4). In addition, an Evans NMR experiment reveals a magnetic moment of 5.7 µ_B_ (Supplementary Materials Figure S5), very close to that expected for a high-spin Mn^II^ center (5.9 µ_B_). Thus, the EPR and solution magnetic moment confirm that **1** remains mononuclear in solution.

### 2.2. Formation and Characterization of [Mn^III^(OH)(^6Me^dpaq^5NO2^)](OTf) *(****2****)*

Complex **1** reacts rapidly with 0.5 equiv. PhIO, resulting in the formation of a golden amber-colored solution with a single absorption shoulder centered at ca. 550 nm (ε = 226 M^−1^ cm^−1^; see Supplementary Materials Figure S6). We refer to this new species as **2**. Similar spectral changes were observed previously for the reaction of [Mn^II^(OH_2_)(^6Me^dpaq)](OTf) with PhIO. The magnetic moment of **2** (4.8 µ_B_) is consistent with a mononuclear *S =* 2 Mn^III^ center (4.9 µ_B_) (Supplementary Materials Figure S7).

**Figure 4:**
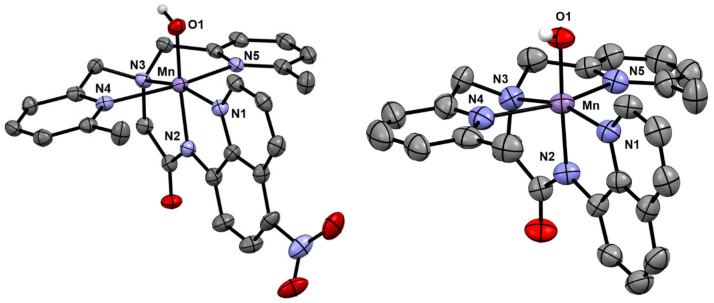
X-ray crystallographic structure of [Mn^III^(OH)(^6Me^dpaq^5NO2^)](OTf) (**left**) and [Mn^III^(OH)(^6Me^dpaq)](OTf) (**right**) [48]. The triflate counter anion and hydrogen atoms of the ^6Me^dpaq^5NO2^ ligand have been removed for clarity.

X-ray diffraction studies of single crystals obtained from **2** establish this species as the mononuclear Mn^III^-hydroxo complex [Mn^III^(OH)(^6Me^dpaq^5NO2^)](OTf). In this complex, a six-coordinate Mn^III^ center is in a pseudo-octahedral geometry with the hydroxo ligand trans to the amide functional group (N2) (Figure 4). This coordination matches with that previously reported for the Mn^III^–hydroxo complexes of dpaq and its derivatives (Table 2). The Mn–O1 bond distance of 1.826(4) Å is just slightly elongated from that observed in [Mn^III^(OH)(^6Me^dpaq)](OTf) and [Mn^III^(OH)(dpaq)](OTf) (1.806(6) and 1.806(13) Å, respectively) and is within the range of the Mn–OH bond lengths reported for other Mn^III^–hydroxo complexes (1.81–1.86 Å). Further bond comparisons with [Mn^III^(OH)(^6Me^dpaq)](OTf) reveal that the addition of the nitro group has perturbed some of the Mn–N bond lengths more than others. Specifically, Mn–N1 and Mn–N2, involving the quinolyl and amide groups, respectively, remain largely unchanged within error. In contrast, Mn–N3 of **2** has contracted by ca. 0.02 Å relative to [Mn^III^(OH)(^6Me^dpaq)](OTf), while Mn–N4 has elongated by 0.075 Å and Mn–N5 has contracted by 0.039 Å. 

**Figure 5:**
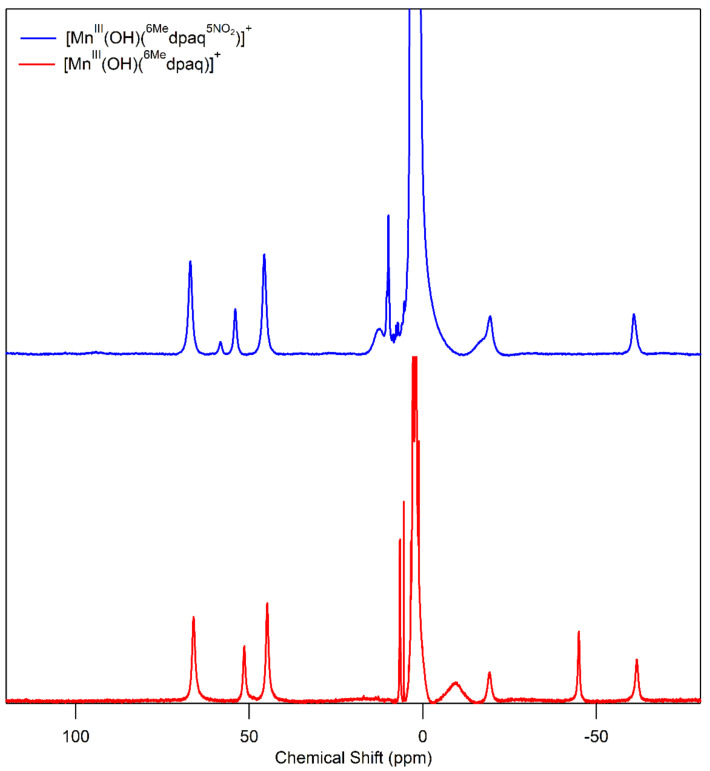
^1^H NMR spectra of **2** (**top**) and [Mn^III^(OH)(^6Me^dpaq)]^+^ (**bottom**) [48] in CD_3_CN at 298 K.

We used ^1^H NMR spectroscopy to gain further insight into the solution structure of **2** in MeCN. The high-spin Mn^III^ center in **2** caused protons near the metal ion to show broadened and shifted ^1^H NMR resonances (Figure 5). On the basis of a detailed analysis of the ^1^H NMR spectrum of the related [Mn^III^(OH)(dpaq)]^+^ complex [52], we provide tentative assignments for some of the resonances of **2** in Table 2. The ^1^H NMR spectrum of **2** in CD_3_CN shows nine broad peaks (Figure 5) spanning the chemical shift range of 67 to −61 ppm (Table 3). There is a cluster of four downfield peaks from 67 to 45 ppm, two peaks near 10 ppm, and three upfield peaks at −16.8 (shoulder), −19.4, and −60.7 ppm. The spectrum bears a strong resemblance to that of [Mn^III^(OH)(^6Me^dpaq)](OTf), which is included in Figure 5 (bottom) for comparison. Specifically, the ^1^H NMR spectrum of [Mn^III^(OH)(^6Me^dpaq)](OTf) shows three peaks in the downfield region of 67 to 45 ppm, and the chemical shifts for these peaks are nearly identical to three of the downfield peaks of **2** (Table S1). In addition, the upfield region of the ^1^H NMR spectrum of [Mn^III^(OH)(^6Me^dpaq)](OTf) shows a broad peak at −9.6 ppm, which corresponds well to the broad peak of **2** at −17.1 ppm, and the spectrum of [Mn^III^(OH)(^6Me^dpaq)](OTf) has two sharper peaks at −19.3 and −61.6 ppm, which are reproduced in the ^1^H NMR spectrum of **2** at −19.4 and −60.7 ppm. The [Mn^III^(OH)(^6Me^dpaq)](OTf) complex has an additional upfield peak at −45.0 ppm that is clearly absent in the ^1^H NMR spectrum of **2** (Figure 5, top and bottom). We attribute this peak in [Mn^III^(OH)(^6Me^dpaq)](OTf) to the 5-quinolyl proton, as this proton has been substituted by the nitro group in **2**. In support, the ^1^H NMR spectrum of [Mn^III^(OH)(dpaq)](OTf) has a proton resonance at −33.7 ppm that was lacking in the spectrum of the nitro-substituted [Mn^III^(OH)(dpaq^5NO2^)](OTf) complex [52].

### 2.3. Formation of [Mn^III^(OO^t^Bu)(^6Me^dpaq^5NO2^)]^+^ *(****3a****)* and [Mn^III^(OOCm)(^6Me^dpaq^5NO2^)]^+^ *(****3b****)*

The addition of 1.5 equiv. of *^t^*BuOOH to the Mn^II^ complex **1** in CH_3_CN at 298 K results in the formation of a green solution (**3a**) with a prominent electronic absorption feature at 665 nm (ε = 231 M^−1^ cm^−1^) (Supplementary Materials Figure S8). (The extinction coefficient was determined by assuming a full conversion took place under these conditions.) A similar reaction occurs upon the addition of CmOOH to **1** in CH_3_CN, with the feature occurring as a shoulder at ca. 650 nm (ε = 170 M^−1^ cm^−1^), resulting in an amber-green solution (**3b**) (Supplementary Materials Figure S8).

**Figure 6:**
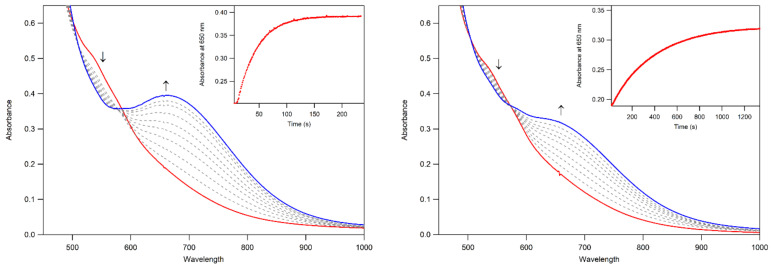
UV–Vis spectra showing the formation of **3a** (**left**) and **3b** (**right**) from the reaction of 3 mM of **2** with 1 equiv. of *^t^*BuOOH (**left**) and CmOOH (**right**) in CH_3_CN at 323 K. The absorbance at 650 nm over time for each reaction is shown inset. The down arrows indicate disappearance of **2** (red trace) and the up arrows indicated formation of **3a** and **3b** (blue traces).

In addition to this method of formation, both **3a** and **3b** can be formed by first generating **2** from the reaction of **1** and 0.5 equiv. PhIO, followed by the addition of 1.0 equiv. of the alkyl hydrogen peroxide (Figure 6). In this method, the formation of each complex is accompanied by isosbestic behavior, indicating the lack of any accumulating intermediate. Similar behavior was observed in the formation of [Mn^III^(OO*^t^*Bu)(^6Me^dpaq)]^+^ from the reaction of the corresponding Mn^III^–hydroxo complex with *^t^*BuOOH and CmOOH [48]. The solution-phase magnetic moments of **3a** and **3b** (4.5 and 4.9 µ_B,_ respectively) were determined using the Evans NMR method (Supplementary Materials Figure S9). These values are consistent with the *S =* 2 Mn^III^ centers, confirming both that these complexes are mononuclear in solution and that the reaction of **2** with alkylhydroperoxides does not result in the oxidation of the Mn^III^ center. ESI-MS data, collected for **3a** and **3b,** reveal prominent peaks with a *m*/*z* of 599.18 and 661.20, respectively, which are consistent with the expected *m*/*z* of [Mn^III^(OO*^t^*Bu)(^6Me^dpaq^5NO2^)]^+^ (calculated *m*/*z* of 599.18) and [Mn^III^(OOCm)(^6Me^dpaq^5NO2^)]^+^ (calculated *m*/*z* of 661.20; see Supplementary Materials Figures S12 and S13).

### 2.4. Spectroscopic Characterization of ***3a*** and ***3b***

**Figure 7:**
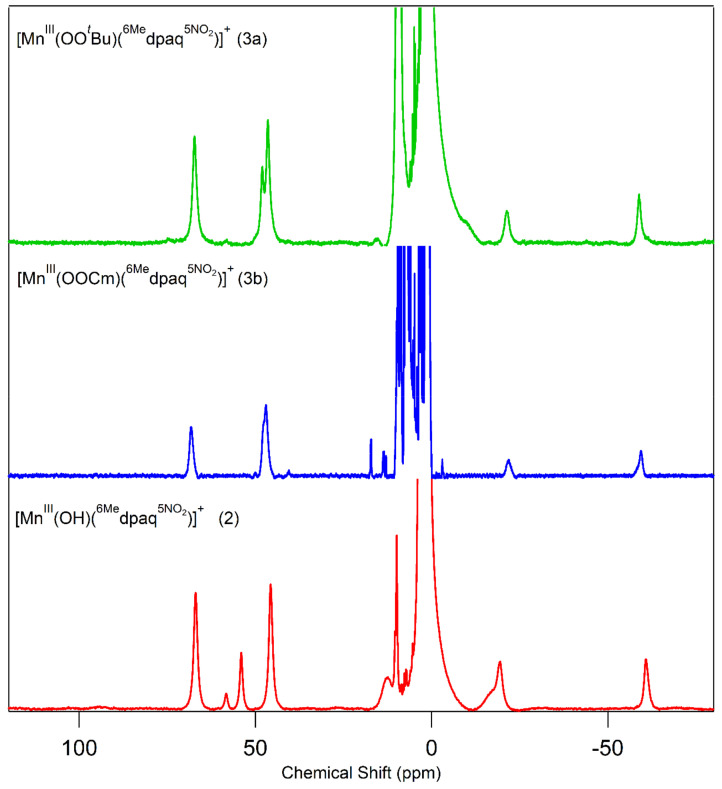
^1^H NMR spectra of **2** (red trace), **3a** (green trace), and **3b** (blue trace) in CD_3_CN at 298 K.

Because we were unable to obtain crystals suitable for X-ray diffraction for either Mn^III^–alkylperoxo complex, we used ^1^H NMR spectroscopy to aid in characterizing the structures of these complexes. The ^1^H NMR spectra of **3a** and **3b** show a set of three downfield peaks in the range of 67 to 45 ppm and two upfield peaks at −21 and −59 ppm (Figure 7 and Table 3). The broad peak at 47.0 ppm for **3b** has a shoulder at 47.6 ppm, suggesting the presence of two closely spaced resonances. (The ^1^H NMR spectrum of **3b** also shows a set of sharp peaks in the 20—0 ppm region, which we attribute to a small amount of free ligand. The previously reported [Mn^III^(OH)(dpaq^5NO2^)]^+^ complex showed similar peaks in the ^1^H NMR spectrum in the presence of D_2_O. We speculate that water, formed as a product of the reaction of [Mn^III^(OH)(dpaq^5NO2^)]^+^ and CmOOH, causes the appearance of a small amount of free ligand in this case.) Although the ^1^H NMR spectra of both **3a** and **3b** have fewer peaks than **2**, the peaks that are observed are at positions similar to the resonances of **2**. Specifically, the upfield peaks of **3a** and **3b** (at ca. −21 and −59 ppm) have chemical shifts nearly identical to the upfield peaks of **2** (ca. −19 and −60 ppm). In addition, the three downfield peaks of **3a** and **3b** at ca. 67, 48, and 46 ppm are at similar positions as the corresponding resonances in **2** (67, 55, and 46 ppm). The similarities in the ^1^H NMR spectra of **3a** and **3b** with that of **2** are consistent with the ^6Me^dpaq^NO2^ ligand having a similar coordination mode for the Mn^III^–alkylperoxo and Mn^III^–hydroxo complexes.

The ^1^H NMR spectra of **3a** and **3b** also bear a strong resemblance to the corresponding spectra of the Mn^III^–alkylperoxo complexes [Mn^III^(OO*^t^*Bu)(^6Me^dpaq)]^+^ and [Mn^III^(OOCm)(^6Me^dpaq)]^+^ (Supplementary Materials Figure S14). The ^1^H NMR spectra for the [Mn^III^(OO*^t^*Bu)(^6Me^dpaq)]^+^ and [Mn^III^(OOCm)(^6Me^dpaq)]^+^ complexes showed three downfield peaks from 67 to 45 ppm and two upfield peaks at −47 and −60 ppm (Supplementary Materials Figure S14). The major difference between these spectra and those of **3a** and **3b** is the upfield signal at −47 ppm in the spectra of [Mn^III^(OO*^t^*Bu)(^6Me^dpaq)]^+^ and [Mn^III^(OOCm)(^6Me^dpaq)]^+^ that is lost in the spectra of **3a** and **3b**. This difference arises because of the substitution of the 5-quinolyl proton in the former complexes with the nitro group in the latter complexes.

**Figure 8:**
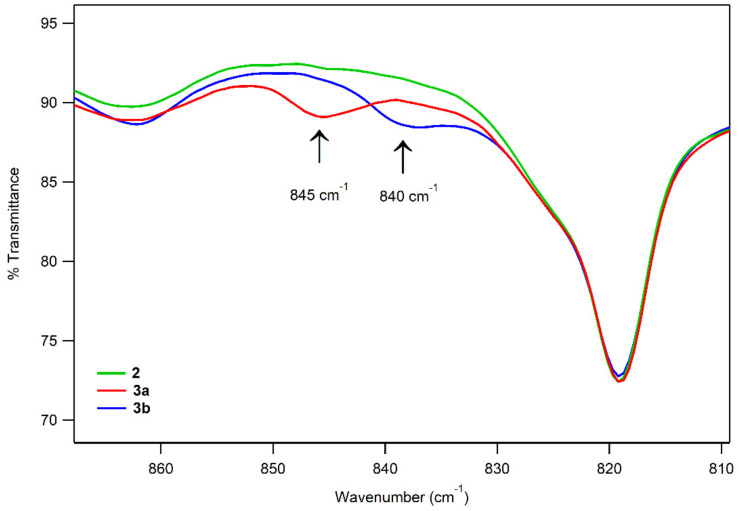
Solution FT-IR spectra of 2 mM solutions of **2** (green), **3a** (red), and **3b** (blue).

The solution FT-IR spectra of **3a** and **3b** show features at 845 cm^−1^ and 840 cm^−1^, respectively, that are absent in the FT-IR spectrum of **2** (Figure 8). These features have energies similar to those of the O–O vibrations previously reported for Mn^III^–alkylperoxo complexes (850–895 cm^−1^) [44,45,47]. The specific O–O vibrational energies for **3a** and **3b** are ~20–30 cm^−1^ lower than those of the related complexes [Mn^III^(OO*^t^*Bu)(^6Me^dpaq)]^+^ and [Mn^III^(OOCm)(^6Me^dpaq)]^+^ (861 and 875 cm^−1^) [48]. The vibrational energies are closest to that reported for an alkoxide-ligated Mn^III^–OO*^t^*Bu complex (850 cm^−1^) [46]. As emphasized in that study, coupling of the O–O stretch with other vibrational modes can make it difficult to relate O–O vibrational energies to either the peroxo bond length or thermal stability. Indeed, in the cases of **3a** and **3b**, we observed a relatively low energy O–O vibration for complexes with high thermal stability.

The EPR analyses of the frozen solutions of **3a** and **3b** in 1:1 CH_3_CN:toluene, collected at 10 K, showed only weak signals in the perpendicular mode (near *g* = 2) and no signals in the parallel mode (Supplementary Materials Figure S15). This is consistent with the lack of X-band signals for many Mn^III^ complexes due to the moderate-to-large zero-field splitting relative to the microwave energy. The weak signals near *g* = 2 are attributed to a minority of Mn^II^ species in the solution. In support, the spin quantification [53] of the frozen solutions of **3a** and **3b** in CH_3_CN showed that the *g* = 2 signals account for only ~15 and 10%, respectively, of the Mn in solution (Supplementary Materials Figures S15 and S16).

### 2.5. DFT Structures of ***3a*** and ***3b***

**Figure 9:**
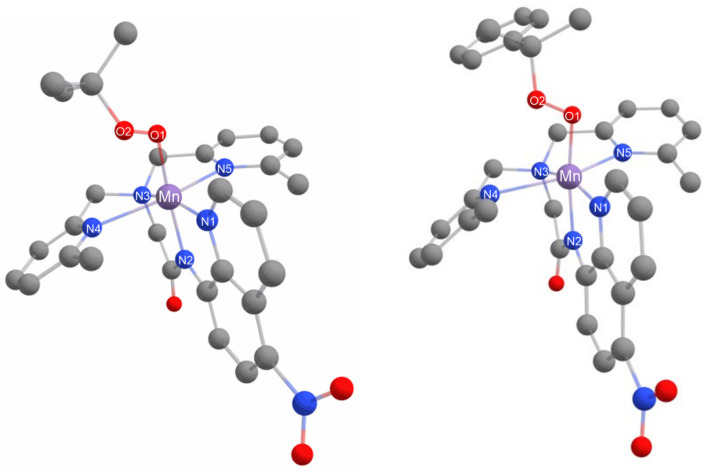
DFT-optimized structures of **3a** (**left**) and **3b** (**right**).

We used DFT computations to predict the structures of **3a** and **3b** The optimized structures are show in Figure 9 and selected metric parameters are in Table 4. In the DFT-optimized structures of **3a** and **3b**, the *tert*-butylperoxo and cumylperoxo ligands are bound *trans* to the amide nitrogen (N2) at N2–Mn–O1 angles of 176.4° and 173.9°, respectively. The Mn–O1 bond lengths for both **3a** and **3b** (1.83 Å) are the same as that of the Mn^III^–hydroxo complex (1.826(4) Å). The average of the Mn–N(pyridine) distances (Mn–N_Ave:4,5_) of **3b** (2.33 Å) is essentially the same as that of the previously reported [Mn^III^(OOCm)(^6Me^dpaq)]^+^ complex (2.34 Å), while that of **3a** is slightly longer (2.35 Å). The oxygen–oxygen bond (O1–O2) for both complexes is 1.45 Å, which is consistent with that determined from the X-ray crystal structure for [Mn^III^(OOCm)(^6Me^dpaq)]^+^ (1.47 Å), and is only slight shorter than the range of values determined for the [Mn^III^(OOCm)(N4S)]^+^ complexes (1.457(5) to 1.51(2) Å). For the DFT structure, projections of the peroxo O1–O2 bond onto the equatorial plane show the bond bisecting the N1–Mn–N4 bond angle, with the **3b** O1–O2 bond being closer to the Mn–N4 bond than in the **3a** complex. The Mn–O1–O2 bond angle of **3a** (107.3°) is smaller than that of both the [Mn^III^(OOCm)(^6Me^dpaq)]^+^ (110.4(2)°) complex and **3b** (114.2°).

### 2.6. Thermal Decay Pathways of ***3a*** and ***3b***

**Figure 10:**
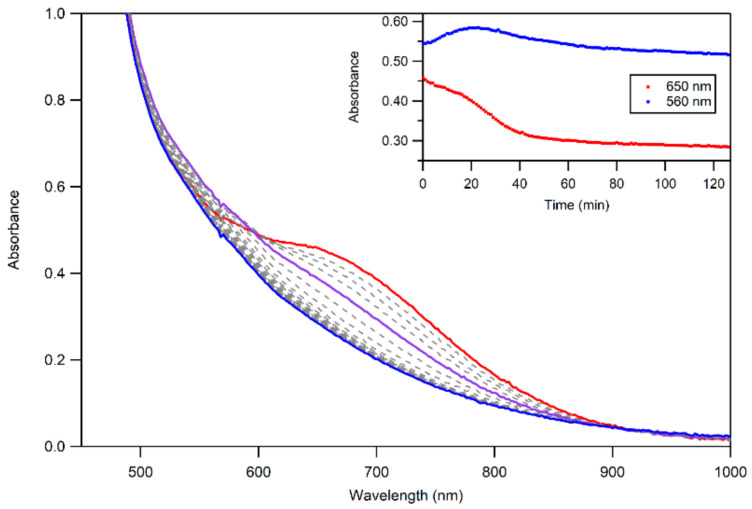
UV–Vis spectra of the thermal decay of **3b** at 323 K. The initial spectrum is shown in red, and the final spectrum is shown in blue—the formation of 2, as an intermediate, is highlighted in purple.

The thermal decay of **3a** and **3b** in CH_3_CN resulted in the loss of intensity of the 650 nm feature associated with the Mn^III^–alkylperoxo complex (Figure 10). The half-lives of the 1.25 mM solutions of **3a** and **3b** are ca. 4 and 3 days, respectively, in CH_3_CN at 298 K. Given this robust thermal stability, our analyses of the decay products of **3a** and **3b** were facilitated by experiments at 323 K. Both **3a** and **3b** decay to give a final spectrum that shows a steady rise from ~900–400 nm with no clear absorption maxima. The final spectra resemble neither that of **2** nor that expected for the Mn^II^ products. This final decay product is thus different from that observed for the [Mn^III^(OOR)(^6Me^dpaq)]^+^ complexes. In that case, the Mn^III^–alkylperoxo complexes underwent thermal decay to re-form the Mn^III^–hydroxo complexes. A close inspection of the thermal decay profiles of **3a** and **3b** shows evidence for the formation of the Mn^III^–hydroxo complex **2**, but, in this case, **2** is an intermediate. For example, we observed a rise in absorption at 560 nm (characteristic of **2**) for the first ~30 min of decay at 323 K (Figure 10, inset). However, **2** is not stable under these conditions and decays after ~40 min. Further support for the presence of the Mn^III^–hydroxo complex **2** as an intermediate in this process is provided by the isolation of X-ray diffraction-quality single crystals of **2** from a solution of **3b** that was stored at 258 K for over 8 weeks. Due to the complexity of the thermal decay process, we were not able to fit the thermal decay profiles into a standard kinetic model.

We also examined the decay products of **3a** and **3b** by EPR spectroscopy. A frozen-solution EPR sample of the final thermal decay products of 3 mM **3b** was prepared by allowing the complex to decay at 343 K in CH_3_CN, then drying and redissolving in 1:1 CH_3_CN:toluene. The EPR spectra, collected at 7 K, reveal an intense signal at g = 2.01, characteristic of a mononuclear Mn^II^ species (Supplementary Materials Figure S18). A quantitative analysis of the EPR signal intensity showed that the *g* = 2.01 signal from a Mn^II^ species accounts for ca. 70% of the Mn in solution. Combined with the findings from UV–Vis spectroscopy, this result suggests that there is a partial conversion of **3b** to Mn^II^ products and a partial generation of another product that gives rise to the broad absorption signal (Figure 10). A similar quantitative EPR analysis of a frozen solution of decay products from the thermal decay of **3a** revealed that the EPR-active Mn^II^ species accounted for ca. 34% of the Mn in solution (Supplementary Materials Figure S19).

**Figure 11:**
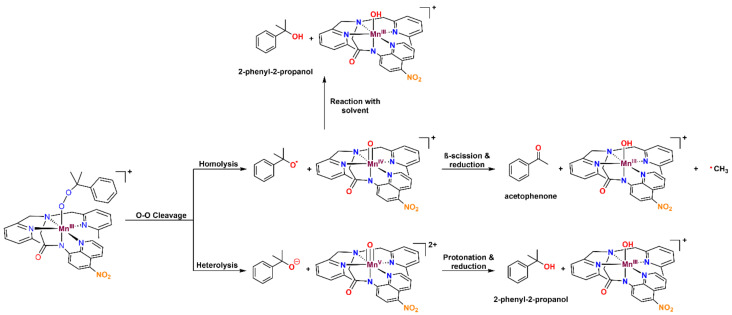
Possible decay pathways for **3b**.

The organic products formed by the decay of metal–cumylperoxo complexes are often used to probe the relative ratios of O–O homolysis and heterolysis. Homolysis yields the cumyloxyl radical, which decays by β-scission to give acetophenone (Figure 11). Heterolysis gives cumyl oxyanion, which can be protonated to form 2-phenyl-2-propanol (cumyl alcohol). The product analysis, following the thermal decay of **3b** in CH_3_CN at 298 K (Figure 11 and Table 5), revealed 49.7 ± 3.5% 2-phenyl-2-propanol and 35.2 ± 2.8% acetophenone relative to the initial concentration of **3b**. The recovery of 85% of the cumylperoxo decay products suggests that O–O cleavage is the dominant decay pathway. The high-valent Mn–oxo species generated by the O–O cleavage is likely quite reactive and rapidly decays to give the mixture of Mn products. The organic products of the thermal decay of **3a** were not quantified due to the volatility of acetone, one of the potential products, which renders quantification unreliable.

The ~50:35 2-phenyl-2-propanol/acetophenone ratio for **3b** marks a ~10% increase in the product ratio compared to [Mn^III^(OOCm)(^6Me^dpaq)]^+^ (61:26 2-phenyl-2-propanol: acetophenone; see Table 5). However, the relationship of metal–cumylperoxo decay products to O–O homolysis versus heterolysis in CH_3_CN is complicated by the reaction of the cumyloxyl radical with solvent. Specifically, the β-scission of the cumyloxyl radical must compete with the cumyloxyl radical abstracting a hydrogen atom from solvent. This latter reaction leads to the formation of 2-phenyl-2-propanol from O–O homolysis (Figure 11). Because the reaction between the cumyloxyl radical and solvent is much slower in a deuterated solvent, we analyzed the decay of **3b** in CD_3_CN. The decay of **3b** at 298 K in CD_3_CN resulted in 24.0 ± 1.6% 2-phenyl-2-propanol and 71.5 ± 4.3% acetophenone relative to the initial **3b** concentration (Table 5). This shift in product distribution from 50:35 2-phenyl-2-propanol/acetophenone in CH_3_CN to 24:72 2-phenyl-2-propanol/acetophenone in CD_3_CN suggests that a substantial fraction of the 2-phenyl-2-propanol produced by the decay of **3b** in CH_3_CN was generated by the reaction of the cumuloxyl radical with solvent. However, we still observed a different 2-phenyl-2-propanol/acetophenone ratio for **3b** than for the previously reported [Mn^III^(OOCm)(^6Me^dpaq)]^+^ (71:24 and 40:50, respectively). Thus, the inclusion of the nitro substituent in **3b** appears to shift the O–O homolysis/heterolysis ratio. Because the nitro group weakens the donation from the amide ligand *trans* to the alkylperoxo group, this result illustrates that subtle changes in the donor properties of a *trans* ligand can have a noticeable effect on the relative energies of the O–O cleavage pathways.

### 2.7. Oxidation of TEMPOH by the Mn^II^–Hydroxo Complex 2

**Figure 12:**
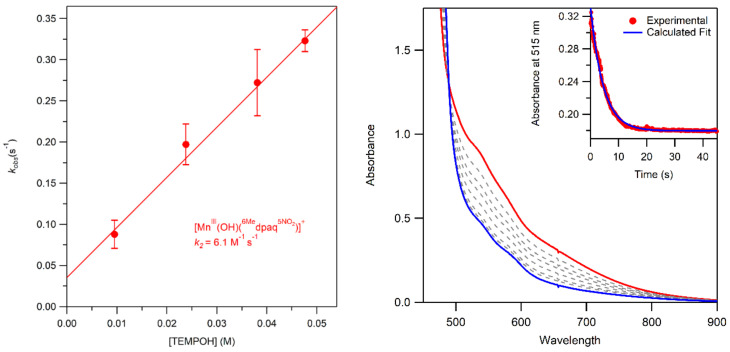
Pseudo-first-order rate constants as a function of [TEMPOH] for a solution of **2** at 238 K in CH_3_CN (**left**) and UV–Vis spectra of the reaction of **2** (3.00 mM) with 10 equiv. TEMPOH at 238 K in CH_3_CN (**right**). Example of pseudo-first-order fit calculation for 1.00 mM solution of **2** with 25 equiv. TEMPOH at 238 K in CH_3_CN (**right inset**).

The addition of 10–50 equiv. TEMPOH to an anerobic solution of **2** (3 mM in CH_3_CN) at 238 K resulted in the rapid loss of intensity of the broad feature ca. 550 nm (Figure 12, right). The resulting final spectrum resembles that of **1**, but the absorption signal is too intense to be from **1** alone. The same final spectrum is observed no matter how much TEMPOH is added. Analysis of the product solution via ^1^H NMR shows no signals associated with **2**, suggesting that the products are a mixture of **1** and other Mn^II^ complexes (Supplementary Materials Figure S20). The decay of **2** upon the addition of TEMPOH could be well-fitted using a first-order model (Figure 12, right inset), yielding a pseudo-first-order rate constant (*k*_obs_). A linear fit of *k*_obs_ versus TEMPOH concentration (Figure 12, left) yielded a second-order rate constant for the reaction of TEMPOH with **2** of 6.1 M^−1^s^−1^ at 298 K in CH_3_CN. This rate fits well with those of similar Mn^III^–hydroxo complexes, including [Mn^III^(OH)(dpaq^5NO2^)]^+^ (7 M^−1^s^−1^) [52] and [Mn^III^(OH)(^6Me^dpaq)]^+^ (3.4 M^−1^s^−1^) [54].

### 2.8. Oxidation of Triphenylphosphine by Mn^III^–Alkylperoxo Complexes

**Figure 13:**
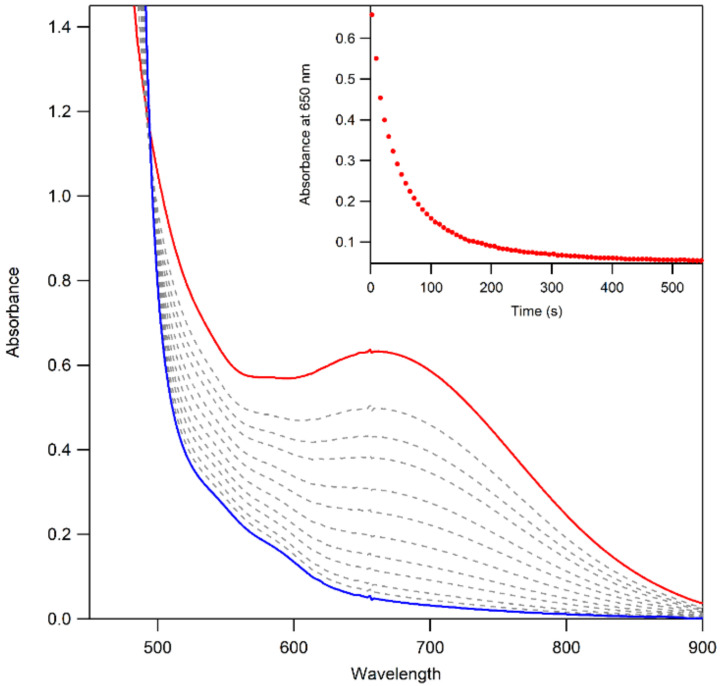
UV–Vis spectra of the reaction of a 3 mM solution of **3a** in acetonitrile (red) with 100 equiv. triphenylphosphine at 298 K. The final spectrum is shown in blue. The absorbance at 650 nm over time is shown in the inset.

The addition of 100 equiv. PPh_3_ to a solution of **3a** (1.00 mM in CH_3_CN) at 298 K resulted in the loss of intensity of the electronic absorption feature at 660 nm over the course of ca. 6 min (Figure 13). Further analysis of the product solution via ^1^H NMR showed only a trace amount of signals consistent with those of **2** or **3a**, indicating that the reaction yields a mixture of **1** and other Mn^II^ products. The decay of the 660 nm absorption signal was unable to be fit with a first-order model, and, as such, we were unable to obtain a pseudo-first-order rate constant or second-order rate constant for the reaction (see Supplementary Materials for more details regarding the complexity of this reaction). While we were unable to obtain a rate constant for the reaction of **3a** with PPh_3_, the *t_1/2_* of the reaction (~40 s) was approximately 16 times faster than that reported for the reaction of [Mn^III^(OO*^t^*Bu)(^6Me^dpaq)]^+^ with PPh_3_ (~650 s). Thus, we observe a dramatic rate enhancement in the reaction of **3a** with PPh_3_, indicating that **3a** is a significantly more electrophilic oxidant. The increased electrophilicity can be rationalized by the nitro substituent in **3a**, which can mitigate the charge donation from the amide to the Mn^III^–OOR unit. A ^31^P NMR analysis of the organic products revealed the formation of Ph_3_PO (Supplementary Materials Figure S22). Quantification of the organic products of the reaction of **3b** with PPh_3_ at 298 K in CH_3_CN via GC-MS revealed a distribution of 67.4 ± 3.7% 2-phenyl-2-propanol and 15.1 ± 2.4% acetophenone based on the initial concentration of the Mn^III^–alkylperoxo complex. The reaction between [Mn^III^(OOCm)(^6Me^dpaq)]^+^ and PPh_3_ was previously characterized in the same fashion and was found to yield a distribution of 88.5 ± 0.3% 2-phenyl-2-propanol to 1.5 ± 0.3% acetophenone [48]. The increase in acetophenone in **3b** compared to the previous complex is consistent with the increased portion of homolytic cleavage observed in the thermal decay.

## 3. Discussion

In order to further probe the effect of the ligand sphere on the reactivity and decay pathways of Mn^III^–alkylperoxo complexes, we developed a new ligand (^6Me^dpaq^5NO2^) that perturbs the electronic properties *trans* to the alkylperoxo unit. This perturbation only minimally affects the thermal stability of the Mn^III^–alkylperoxo complex, as complexes **3a** and **3b** have half-lives in MeCN at room temperature of ~4 days. While Mn^III^–alkylperoxo complexes that are stable at room temperature are still relatively rare, these complexes all contain two common features [44,46,48]. First, the complexes contain at least two N-donor ligands with elongated Mn–N bonds *cis* to the alkylperoxo unit. Second, the complexes lack strong π-acidic ligands *cis* to the alkylperoxo ligand. As we have discussed in a recent computational study, the lack of a π-acidic ligand increases the Mn^III^ quintet-triplet spin gap, which disfavors a two-state mechanism predicted to promote O–O cleavage for both the Mn^III^–alkylperoxo and Mn^III^–hydroperoxo complexes [49].

An analysis of the organic decay products of the Mn^III^–cumylperoxo complex **3b** examined in this work illustrates that *trans* effects also modulate the O–O cleavage pathways of Mn^III^–alkylperoxo complexes. Specifically, the weaker *trans* donor in **3b** leads to an increased production of homolysis reaction products compared to the [Mn^III^(OOCm)(^6Me^dpaq)]^+^ complex (Table 5). While the basis for this change in reaction products is unclear at present, we note that a small change in the relative energies of the reaction barriers for homolysis and heterolysis would account for the observed change in the reaction products.

In contrast to the moderate effect on the O–O cleavage pathways, the new complexes **3a** and **3b** show much faster rates of reaction with the substrate triphenylphosphine. In fact, the rate of reaction with **3a** is sixteen-fold faster than that of the previous [Mn^III^(OO*^t^*Bu)(^6Me^dpaq)]^+^ complex. To date, there are few examples of Mn^III^–alkylperoxo complexes that react directly with substrates, so it is difficult to make general statements about the factors governing reactivity. However, the increase in the rate of triphenylphosphine oxidation by **3a** and **3b** suggests that these complexes are more electrophilic than the previously reported [Mn^III^(OOR)(^6Me^dpaq)]^+^ complexes. We propose that the weaker donation from the *trans* amide ligand causes this increase in the electrophilicity of the Mn^III^–alkylperoxo unit. For comparison, we note that Mn^III^–hydroperoxo complexes have been shown to attack thioanisole and its derivatives, which is also a showcase of electrophilic reactivity [33,34].

When taken as a whole, data for this new series of complexes suggest that it is possible to make alterations in the ligand sphere of Mn^III^–alkylperoxo complexes that allow for the separate tuning of reactivity and stability. Herein, a change to the ligand sphere allowed for (1) a marked increase in reactivity towards an organic substrate, (2) a modest change in the distribution of O–O cleavage products from homolytic and heterolytic pathways, and (3) little change in thermal stability. Such an ability to tune these parameters separately is promising for future study, pointing to the possibility of a complex designed for increased reactivity and O–O heterolytic cleavage while maintaining stability under mild conditions.

## 4. Materials and Methods

### 4.1. General Methods and Instrumentation

All chemicals were used as obtained from commercial sources unless noted otherwise. 2-(bis((6-methylpyridin-2-yl)methyl)amino)-*N*-(quinoline-8-yl)acetamide (H^6Me^dpaq) was synthesized according to a reported procedure [48]. The concentration of *tert*-butyl hydroperoxide (*^t^*BuOOH) in the decane stock solution was found to be 4.3 M by iodometric titration. The experiments were performed under standard atmospheric conditions unless otherwise noted.

The electronic absorption experiments were performed using either a Varian Cary 50 Bio UV–Visible spectrophotometer (Varian, Cranford, NJ, USA) or an Agilent 8453 UV–Visible spectroscopy system (Agilent, Santa Clara, CA, USA). The reaction temperature was controlled using either a Unisoku cryostat (Unisoku, Hirakata, Japan) and stirrer or a Quantum Northwest TC 1 temperature controller and stirrer (Quantum Northwest, Liberty Lake, WA, USA). Electrospray ionization mass spectrometry (ESI-MS) experiments were performed using an LCT Premier MircoMass electrospray time-of-flight instrument (MircoMass, Cary, NC, USA). The X-band EPR experiments were conducted using a Bruker EMXplus with Oxford ESR900 continuous-flow liquid helium cryostat and an Oxford ITC503 temperature system (Bruker, Billerica, MA, USA). ^1^H and ^31^P NMR spectra were obtained using a Bruker DRX 400 MHz NMR spectrometer (Bruker, Billerica, MA, USA). The hyperfine-shifted ^1^H NMR spectra were collected within the spectra width of 150 to −100 ppm with 1000 scans to provide a sufficient S/N. Spectra were baseline subtracted with the multipoint fitting procedure using the spline functions in the MestReNova program. GC analysis was performed on the Shimadzu GCMS-QP2010 SE instrument (Shimadzu, Kyoto, Japan). FT-IR spectra were collected using a Shimadzu IRTracer-100 spectrophotometer on a 1 mm ZnSe permanently sealed cell from Buck Scientific (Norwalk, CT, USA).

### 4.2. Synthesis of (H^6Me^dpaq^5NO2^)

In a round bottom flask, 2.0011 g (0.004866 mol) (H^6Me^dpaq) was dissolved in 100 mL of H_2_SO_4_ and stirred while cooling in an ice bath. A total of 0.5915 g (0.005850 mol) KNO_3_ was added, and the solution was stirred in an ice bath. After 3 h, the solution was neutralized via the addition of ammonium hydroxide solution until a pH of 7 was reached. The resulting solution was extracted with dichloromethane and water, washed with a sodium carbonate solution, and washed with sodium chloride brine. The solvent was removed under vacuum, and the resulting solid was redissolved into acetonitrile. The solvent was removed, yielding a dry powder, which was characterized by ^1^H NMR, ^13^C NMR, and HSQC NMR (Supplementary Materials Figures S1–S3). The final product was obtained as a dark yellow solid with a 96% yield.

### 4.3. Synthesis of Mn^II^(OTf)(^6Me^dpaq^5NO2^) *(****1****)*, [Mn^III^(OH)(^6Me^dpaq^5NO2^)]^+^ *(****2****)*, and [Mn^III^(OOR)(^6Me^dpaq^5NO2^)]^+^ (***3a***: R: ^t^Bu and ***3b***: R: Cm)

The reaction of 0.7501 g (0.001648 mol) H^6Me^dpaq^5NO2^ with 0.789 g (0.001813 mol) Mn^II^(OTf)_2_∙2CH_3_CN was performed in 50 mL of dichloromethane under an inert atmosphere in the presence of 0.158 g (0.001648 mol) of NaO*^t^*Bu as a base. The reaction was stirred for 18 h and yielded a dark orange solution. The solution was passed through a syringe filter, concentrated in vacuo, and layered with diethyl ether. This procedure led to the formation of a yellow-orange precipitate. The solvent was decanted, and the orange was solid dried, redissolved in minimal CH_2_Cl_2_, and layered with diethyl ether. The recrystallization procedure was repeated three more times, and an orange solid was obtained with an 89% yield. Elemental analysis: the carbon expected was 47.35%, we found 47.61%; the hydrogen expected was 3.52%, we found 3.63%; the nitrogen expected was 12.74%, we found 12.87%. The powder X-ray diffraction is shown in Supplementary Materials Figure S23 (left).

In a round bottom flask, 0.6013 g (0.0009118 mol) of Mn^II^(OTf)^6Me^dpaq^5NO2^ was combined with 0.1008 g (0.0004559 mol) of iodosobenzene in 40 mL of CH_2_Cl_2_. The solution was stirred at room temperature for 2 h and then layered with diethyl ether. This procedure led to the formation of a dark green-brown precipitate. The solvent was decanted, and the solid was washed with fresh ether. The solid was then dried, redissolved in a mixture of CH_2_Cl_2_ and acetonitrile (1:4 *v*/*v*), and layered with diethyl ether. This process was repeated one more time, and a green-brown solid was obtained with a 60% yield. The powder X-ray diffraction is shown in Supplementary Materials Figure S23 (right).

The **3a** and **3b** samples were prepared by dissolving **2** in acetonitrile (typically 1.00–5.00 mM) and reacting with a solution of 1 equivalent of the corresponding alkyl hydrogen peroxide in acetonitrile. The reaction was monitored via UV–Vis spectroscopy (typically at 25–60 °C). The full formation of the Mn^III^–alkylperoxo complex was determined by monitoring the feature at ca. 650 nm over time for a plateau in absorbance.

### 4.4. Thermal Decay Reactions of ***3a*** and ***3b*** and Reactivity with PPh_3_

The sample solutions (1.00–3.00 mM) of **3a** and **3b** in CH_3_CN were prepared and transferred to a quartz cuvette. The decay kinetics were monitored on a Varian Cary 50 Bio UV–visible spectrophotometer equipped with a temperature controller and stirrer. Analogous samples were prepared in CH_3_CN and transferred to quartz cuvettes. The cuvettes were then sealed with a septum and sparged with nitrogen gas before undergoing decay kinetic monitoring.

Samples of **3a** and **3b** (1.00–3.00 mM) were prepared in CH_3_CN, transferred into quartz cuvettes, and covered with a rubber septum. A solution of 10–100 equivalents of PPh_3_ per 50–100 uL was prepared using CH_2_Cl_2_. The cuvette was placed in the UV–Vis spectrometer and equilibrated to temperature (25–50 °C) for 5 min before the PPh_3_ solution was added using a gastight syringe. Analogous experiments were conducted using nitrogen-sparged solutions with a nitrogen headspace in the cuvette.

### 4.5. Computational Methods

Electronic structure computations were conducted using the ORCA software program (version 5.0.3) [55,56]. Geometry optimizations and frequency calculations used the TPSSh functional [57] with D3 dispersion corrections [58,59] and def2-SVP as the basis set for carbon and hydrogen. The larger basis set def2-TZVP was used for manganese, nitrogen, and oxygen [60,61]. The default integration grids were used with RIJCOSX integral transformation approximations [62]. Auxiliary basis sets were called using the AutoAux command. All open-shell species were considered at the spin-unrestricted level. Frequency calculations were conducted to confirm that all species were optimized to their minima (no imaginary frequencies).

## Figures and Tables

**Table 1:** Manganese-ligand bond lengths (Å) and angles from X-ray crystal structures of **1** [Mn^II^(OH_2_)(^6Me^dpaq)](OTf) and [Mn^II^(dpaq)](OTf) [51]. *^a^* From ref. [48]; *^b^* from ref. [51].

Bond	1	[Mn^II^(OH_2_)(L)](OTf)	[Mn^II^(L)](OTf)
		*^a^* L = ^6Me^dpaq	*^b^* L = dpaq
Mn–O1	2.169(7)	2.108(3)	2.079(2)
Mn–N1	2.260(6)	2.233(3)	2.214(3)
Mn–N2	2.150(7)	2.152(4)	2.191(3)
Mn–N3	2.281(6)	2.280(3)	2.314(3)
Mn–N4	2.328(7)	2.354(4)	2.244(3)
Mn–N5	2.357(8)	2.417(3)	2.286(3)

**Table 2:** Manganese-ligand bond lengths (Å) and angles from X-ray crystal structures of **2**, [Mn^III^(OH)(^6Me^dpaq)](OTf), [Mn^III^(OH)(dpaq)](OTf), and [Mn^III^Mn^III^(µ-O)(dpaq^5NO2^)_2_](OTf)_2_. *^a^* From ref. [48]. *^b^* From ref. [51]; *^c^* from ref. [52].

Bond	[Mn^III^(OH)(L)](OTf)			[Mn^III^Mn^III^(µ–O)(L)_2_](OTf)_2_
	L = ^6Me^dpaq^5NO2^	*^a^* L = ^6Me^dpaq	*^b^* L = dpaq	*^c^* L = dpaq^5NO2^
Mn–O1	1.826(4)	1.806(6)	1.806(13)	1.7918(4)
Mn–N1	2.043(4)	2.041(7)	2.072(14)	2.054(2)
Mn–N2	1.959(5)	1.962(6)	1.975(14)	1.973(2)
Mn–N3	2.114(4)	2.130(6)	2.173(14)	2.199(2)
Mn–N4	2.397(4)	2.322(6)	2.260(14)	2.186(2)
Mn–N5	2.342(5)	2.381(7)	2.216(15)	2.288(3)

**Table 3:** ^1^H NMR chemical shifts (ppm) for **2**, **3a**, and **3b**. Complexes in CD_3_CN at 298 K.

	2	3a	3b
H-quinoline	67.0	67.3	68.3
NA *^a^*	58.4		
H-pyridine	54.0	48.1	47.6
H-pyridine	45.6	46.5	47.0
NA *^a^*	12.7	9.5	
NA *^a^*	9.9	8.8	
H-pyridine	−16.8		
H-quinoline	−19.4	−21.5	−21.8
H-quinoline	−60.7	−58.9	−59.5

*^a^* Not assigned.

**Table 4:** Manganese-ligand bond lengths (Å) and angles of **3a**, **3b**, and [Mn^III^(OOCm)(^6Me^dpaq)](OTf). Bond lengths and angles of **3a** and **3b** derived from DFT-optimized structures. *^a^* From X-ray structure in ref. [48].

Bond	3a	3b	[Mn^III^(OOCm)(L)](OTf)
	L = ^6Me^dpaq^5NO2^	L = ^6Me^dpaq^5NO2^	*^a^* L = ^6Me^dpaq
Mn–O1	1.833	1.831	1.849(3)
Mn–N1	2.045	2.037	2.044(4)
Mn–N2	1.958	1.950	1.955(4)
Mn–N3	2.153	2.150	2.100(4)
Mn–N4	2.355	2.370	2.284(4)
Mn–N5	2.347	2.296	2.394(4)
O1–O2	1.454	1.451	1.466(4)
Mn–O1–O2	107.3	114.2	110.4(2)
Source	DFT	DFT	X-Ray

**Table 5:** Product distribution obtained via GC-MS for thermal decay of Mn^III^–cumylperoxo complexes in acetonitrile and acetonitrile-*d*_3_. *^a^* From ref. [48].

Complex	Solvent	2-Phenyl-2-propanol	Acetophenone
**3b**	CH_3_CN	49.7 ± 3.5%	35.2 ± 2.8%
*^a^* [Mn^III^(OOCm)(^6Me^dpaq)]^+^	CH_3_CN	61.3 ± 0.1%	25.7 ± 0.1%
**3b**	CD_3_CN	24.0 ± 1.6%	71.5 ± 4.3%
*^a^* [Mn^III^(OOCm)(^6Me^dpaq)]^+^	CD_3_CN	50 ± 0.3%	40 ± 0.3%

## Data Availability

The crystallographic data are available through the Cambridge Crystallographic Data Centre (CCDC) at https://www.ccdc.cam.ac.uk/ with structure codes 2339539, and2339541.

## Supplementary Materials

The following supporting information can be downloaded at: https://www.mdpi.com/article/10.3390/molecules29081849/s1, Synthesis of H^6Me^dpaq^5NO2^, Figure S1: ^1^H NMR spectrum of H^6Me^dpaq^5NO2^, Figure S2: ^13^C NMR spectrum of H^6Me^dpaq^5NO2^, Figure S3: HSQC NMR data for H^6Me^dpaq^5NO2^, Figure S4: Perpendicular-mode EPR spectrum of **1**, Figure S5: Evans NMR spectrum of **1**, Figure S6: UV–Vis spectra of **1** with PhIO, Figure S7: Evans NMR spectrum of **2**, Figure S8: UV–Vis formation of **3a** and **3b**, Figure S9: Evans NMR spectra of **3a** and **3b**, Figure S10: ESI-MS spectrum of **1**, Figure S11: ESI-MS spectrum of **2**, Figure S12: ESI-MS spectrum of **3a**, Figure S13: ESI-MS spectrum of **3b**, Figure S14: ^1^H NMR spectra of **3a**, **3b**, [Mn^III^(OO*^t^*Bu)(^6Me^dpaq)]^+^, and [Mn^III^(OOCm)(^6Me^dpaq)]^+^, Table S1: ^1^H NMR chemical shifts (ppm) for [Mn^III^(OH)(^6Me^dpaq)]^+^, [Mn^III^(OO*^t^*Bu)(^6Me^dpaq)]^+^, and [Mn^III^(OOCm)(^6Me^dpaq)]^+^, Figure S15: X-band EPR spectra of **3a** and **3b**, Figure S16: Perpendicular-mode EPR spectra of **3a**, Figure S17: Perpendicular-mode EPR spectra of **3b**, Figure S18: Perpendicular-mode EPR spectra of **3b** thermal decay products, Figure S19: Perpendicular-mode EPR spectra of **3a** thermal decay products, Figure S20: ^1^H NMR of the products of **2** and TEMPOH, Figure S21: UV–Vis spectra of **2** with PPh_3_, Reaction of **2** with triphenylphosphine, Figure S22: ^31^P NMR of the products of **3a** and PPh_3_, X-ray reports for **1** and **2**, Table S2: Crystal data and structure refinement for **1** and **2** and powder X-ray diffraction (PXRD) instrumentation and methods, Figure S23: PXRD spectra of 1 and 2, DFT structure coordinates for **3a**, DFT structure coordinates for **3b**. Refs. [63,64] are cited in Supplementary Materials.
