# Supplementary material for: Investigating Ligand Sphere Perturbations on MnIII–Alkylperoxo Complexes
Source: Molecules. 2024 Apr 18;29(8):1849. doi: 10.3390/molecules29081849 (PMC11053420; doi:10.3390/molecules29081849)
Supplement: Supplementary file 1 [file molecules-29-01849-s001.zip › molecules-2961488-supplementary.pdf]

Supplementary Information for

Investigating ligand sphere perturbations on Mn^{III}-alkylperoxo complexes

Samuel A. Brunclik, Elizabeth N. Grotemeyer, Zahra Aghaei, Mohammad Rasel Mian, and Timothy A. Jackson *

The University of Kansas, Department of Chemistry and Center for Environmentally Beneficial Catalysis, 1567 Irving Hill Road, Lawrence, KS 66045, USA.

* To whom correspondence should be addressed:
Timothy A. Jackson
Phone: (785) 864-3968
taj@ku.edu

Chracterization of ($H^{6Me}dpaq^{5NO_2}$)

^1H NMR data (500 MHz) for $H^{6Me}dpaq^{5NO}{}_2$ ($CDCl_3$, δ) = 11.86 (s, 1H), 9.25 (dd, *J* = 9.0, 1.9 Hz; 1H), 9.01 (dd, *J* = 4.1, 2.0 Hz; 1H), 8.76 (d, *J* = 8.9 Hz; 1H), 8.47 (d, *J* = 8.9 Hz; 1H), 7.74 (dd, *J* = 8.9, 4.2 Hz; 1H), 7.63 (d, *J* = 7.7 Hz; 2H), 7.50 (dd, *J* = 7.8, 7.7 Hz; 2H), 6.97 (d, *J* = 7.7 Hz; 2H), 3.98 (s, 4H), 3.57 (s, 2H), 2.42 (s, 6H) ppm. ^{13}C NMR data (500 MHz) for $H^{6Me}dpaq^{5NO}{}_2$ ($CDCl_3$, δ) = 170.92 (s, C=O), 158.09 (s, Qu), 157.33 (s, Qu), 148.91 (s, Qu), 140.81 (s, Qu), 138.64 (s, Py), 137.93 (s, Qu), 136.84 (d, Py), 133.22 (s, Qu), 127.87 (s, Qu), 124.64 (s, Qu), 122.02 (s, Py), 120.23 (s, Py), 113.63 (s, Qu), 61.61 ($-CH_2Py$), 59.34 ($-CH_2CO-$), 24.43 (CH_3Py) ppm.

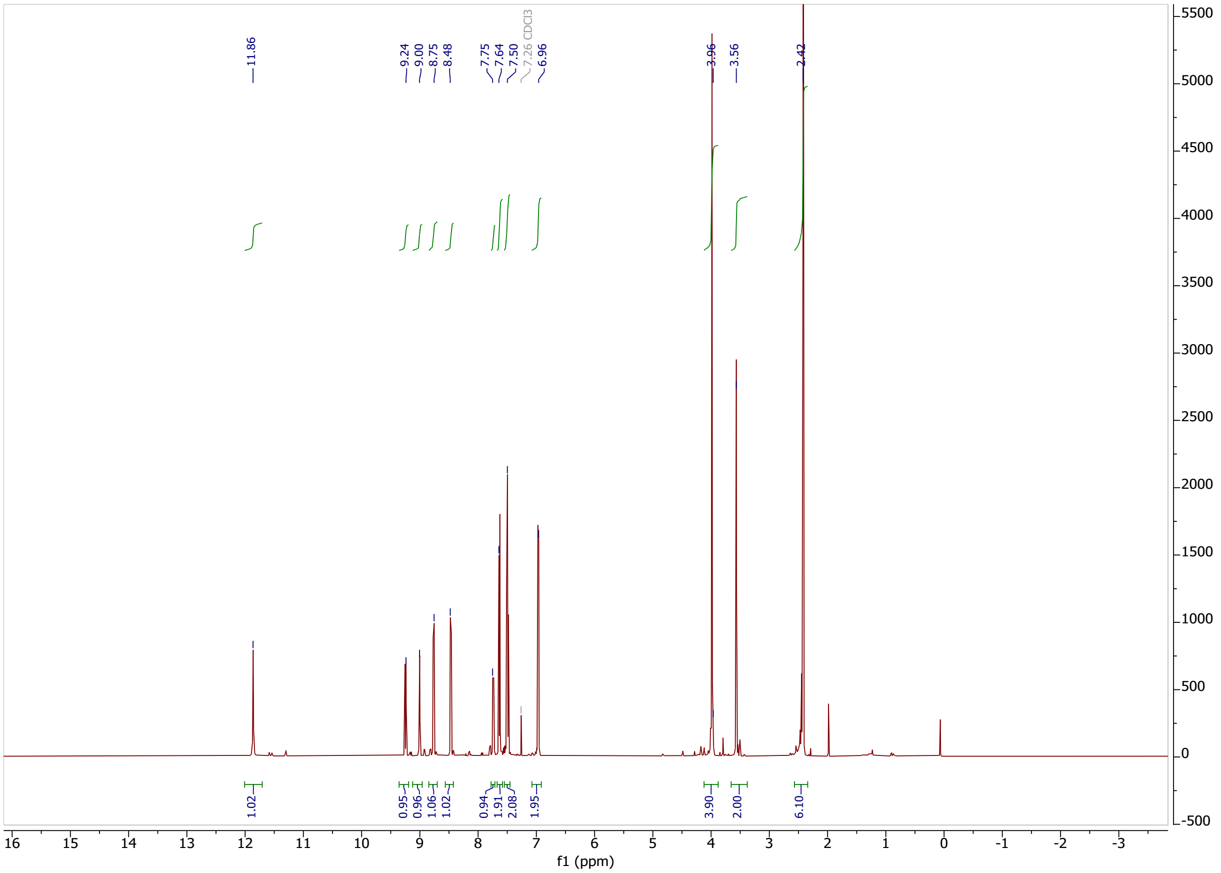

Figure S1. ^1H NMR spectrum of $H^{6Me}dpaq^{5NO_2}$ dissolved in $CDCl_3$ at 298 K.

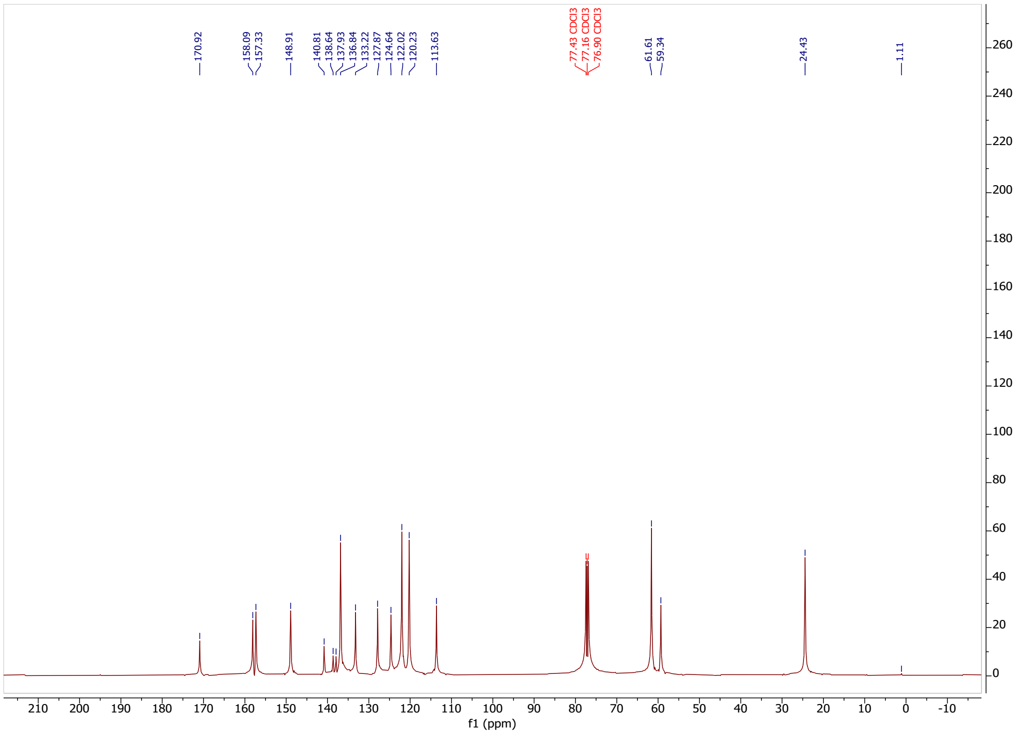

Figure S2. ^{13}C NMR spectrum of H^{6Me}dpaq^{5NO_2} dissolved in $CDCl_3$ at 298 K.

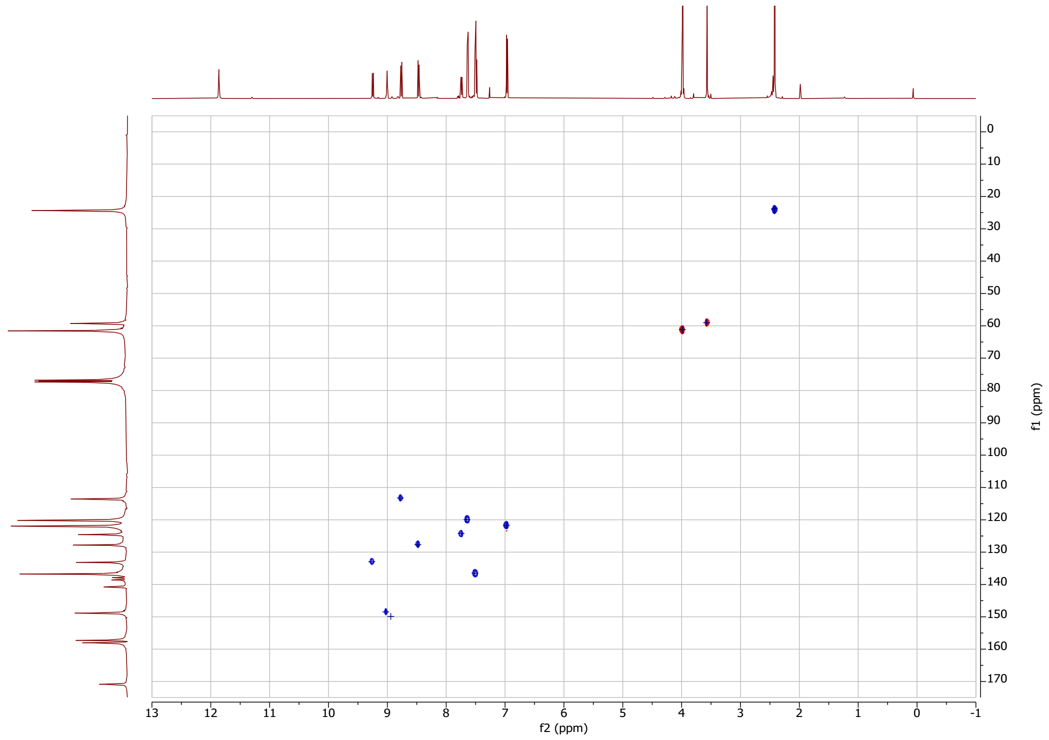

Figure S3. HSQC NMR data for H^{6Me}dpaq^{5NO_2} dissolved in $CDCl_3$ at 298 K.

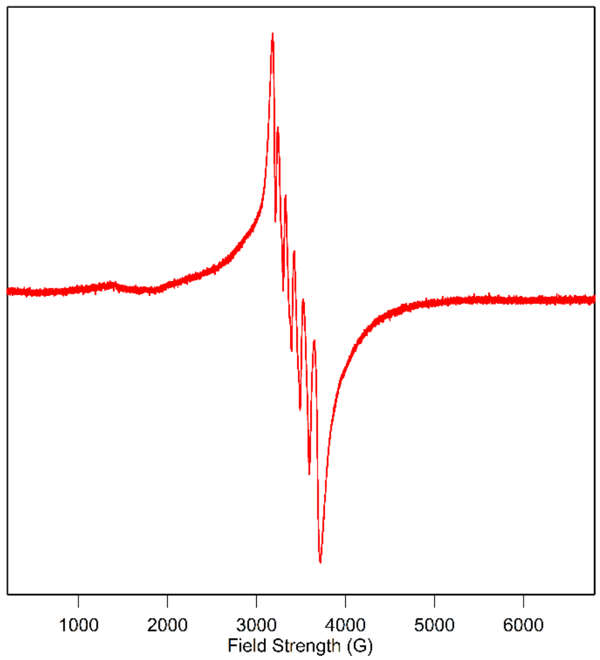

Figure S4. Perpendicular-mode, X-band EPR spectrum of a 2.93 mM solution of **1** dissolved in 1:1 acetonitrile:toluene at 9 K.

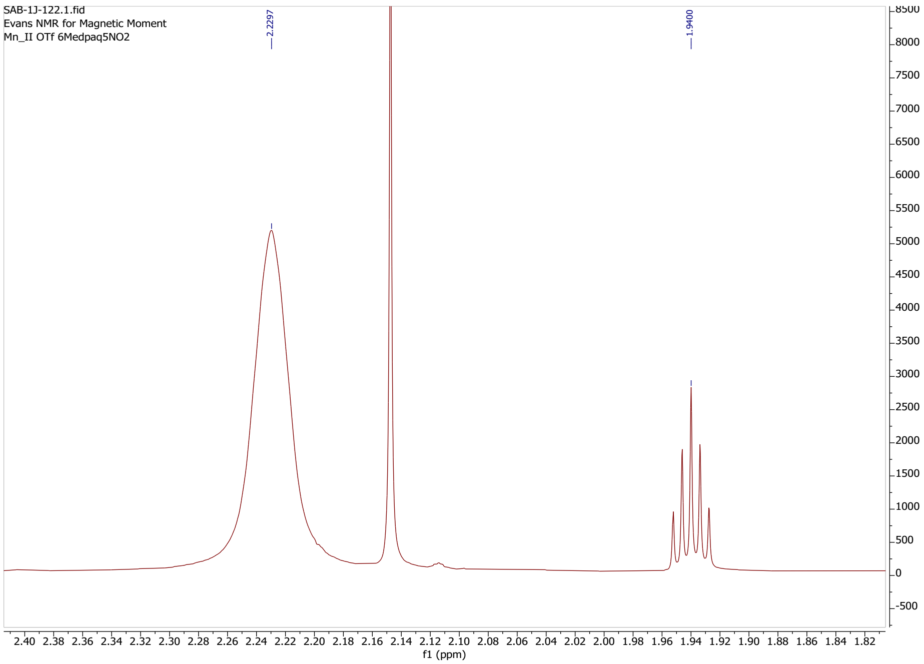

Figure S5. Evans NMR spectrum of **1** in CD_3CN.

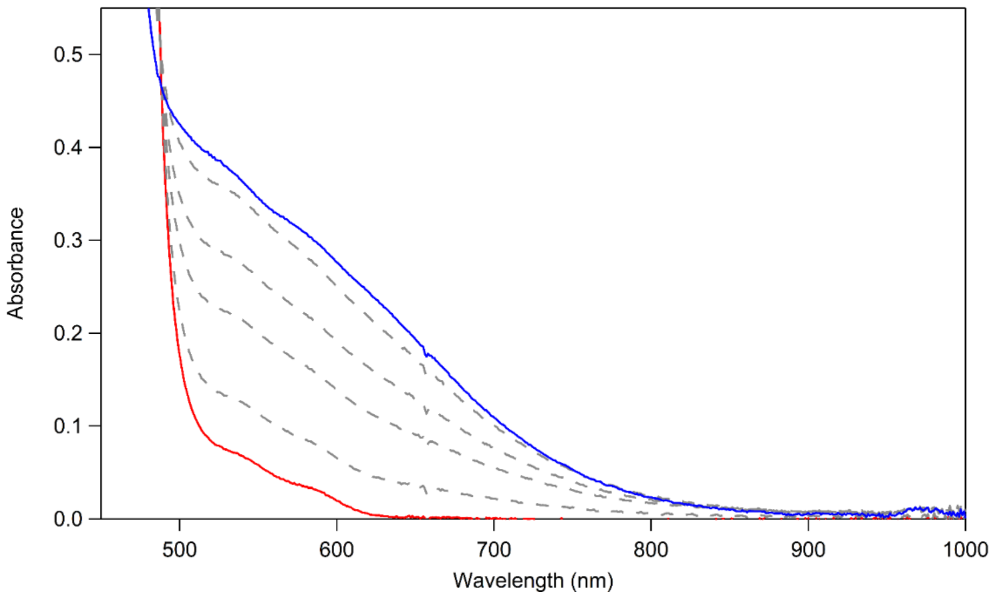

Figure S6. UV-vis spectra of the reaction between of a 1.25 mM solution of **1** and 0.5 equiv. PhIO in CH_3CN at 323 K. The initial spectrum is in red and the final spectrum is blue.

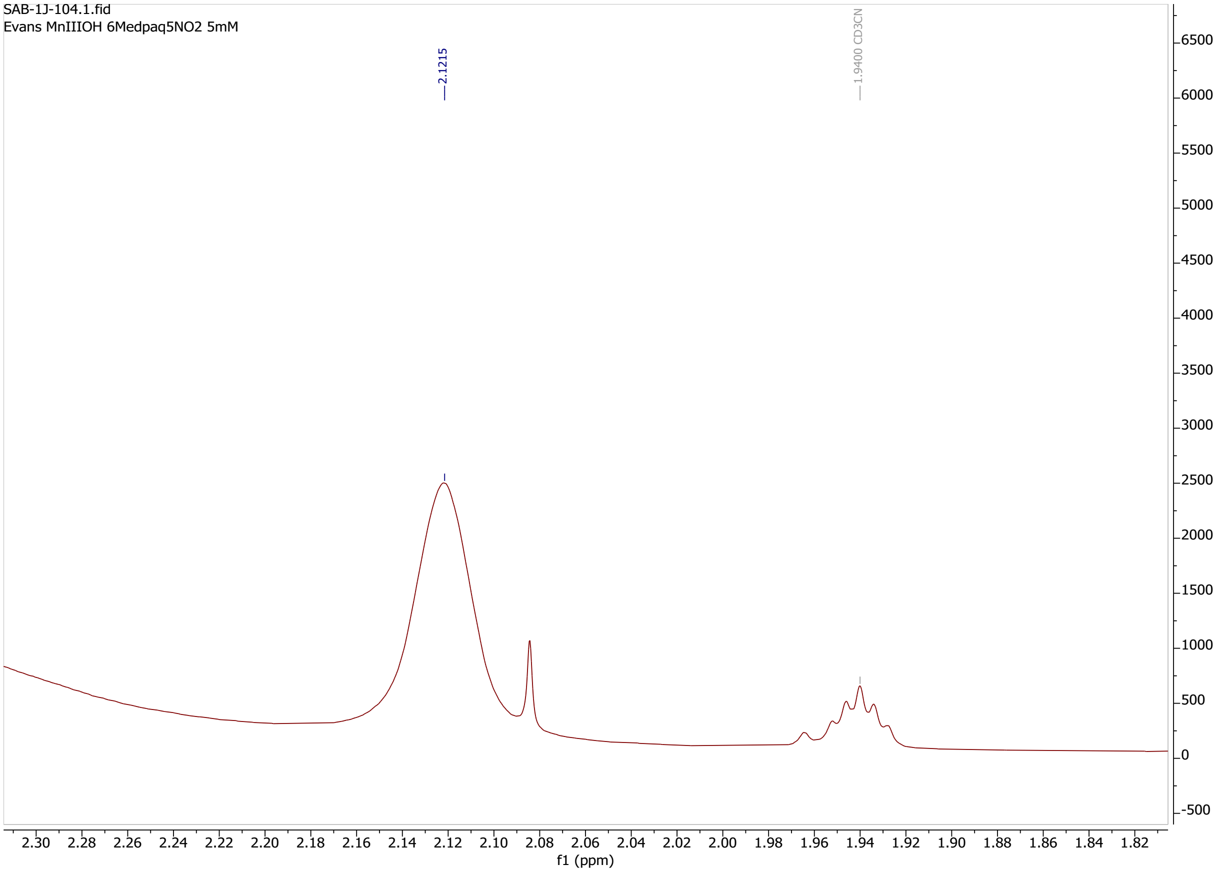

Figure S7. Evans NMR spectrum of **2** in CD_3CN.

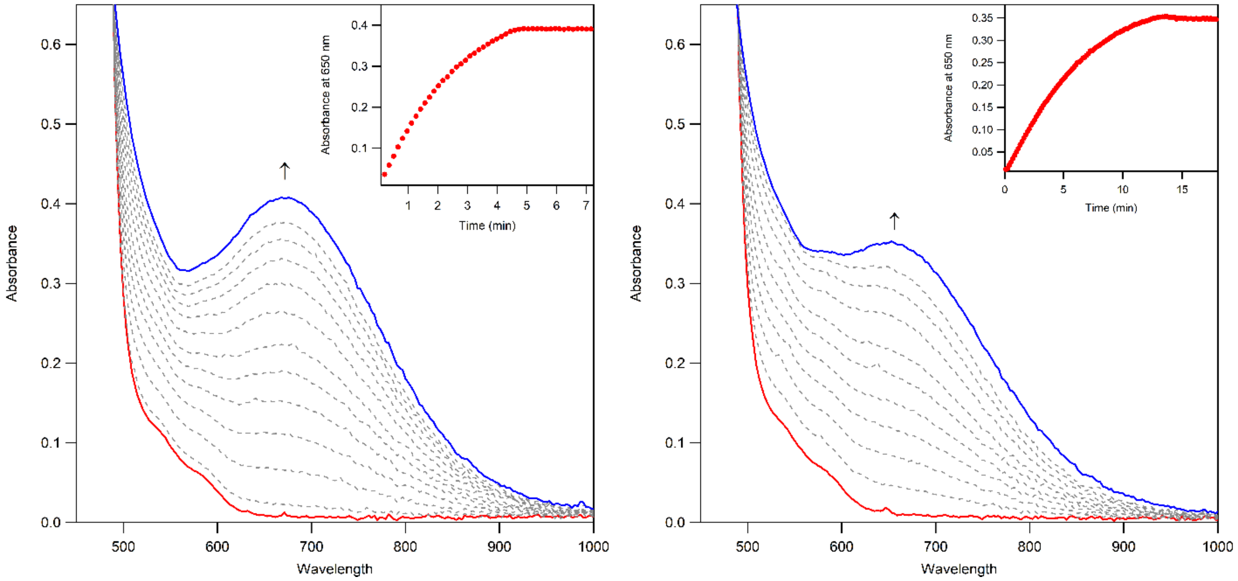

Figure S8. Formation of **3a** (left) and **3b** (right) from the reaction of a 2 mM solution of **1** with 1.5 equiv. ^{t}BuOOH or CmOOH, respectively, at 313 K in atmosphere. The initial spectrum is in red and the final spectrum is blue. Absorbance at 650 nm over time is shown inset.

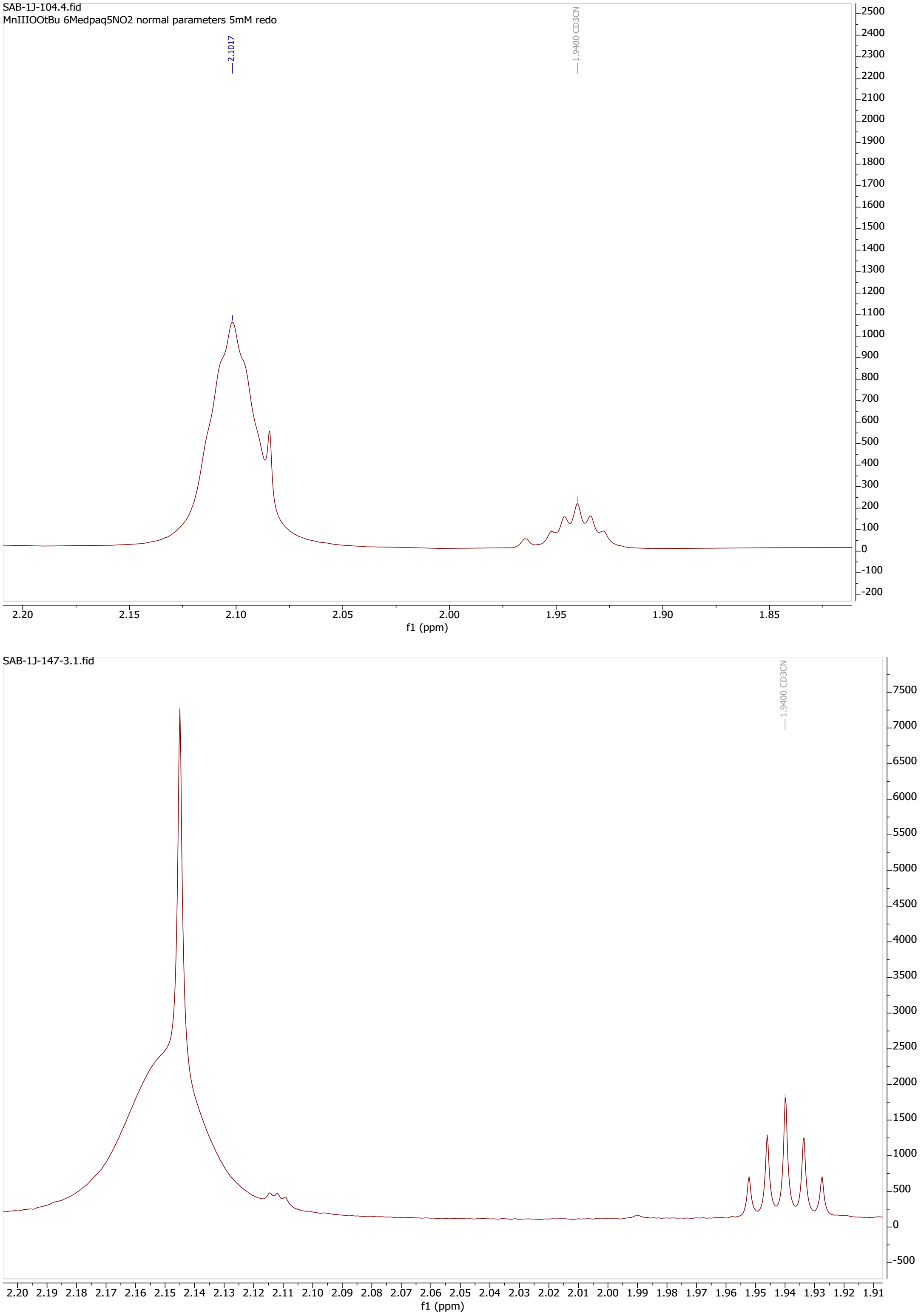

Figure S9. Evans NMR spectrum of **3a** (top) and **3b** (bottom) in CD_3CN.

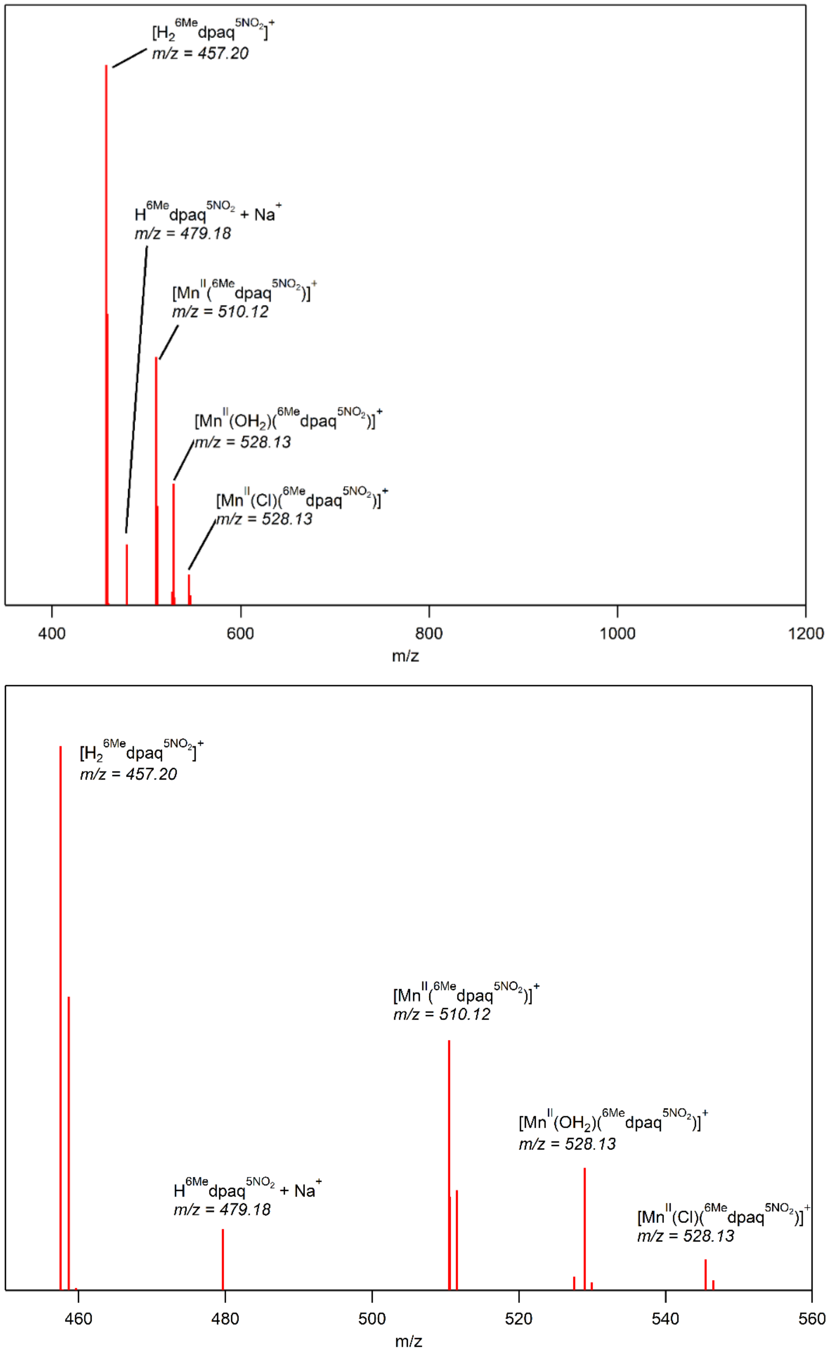

Figure S10. ESI-MS spectrum of **1** in CH_3CN. Both the full spectral region (top) and the region of interest (bottom) are shown.

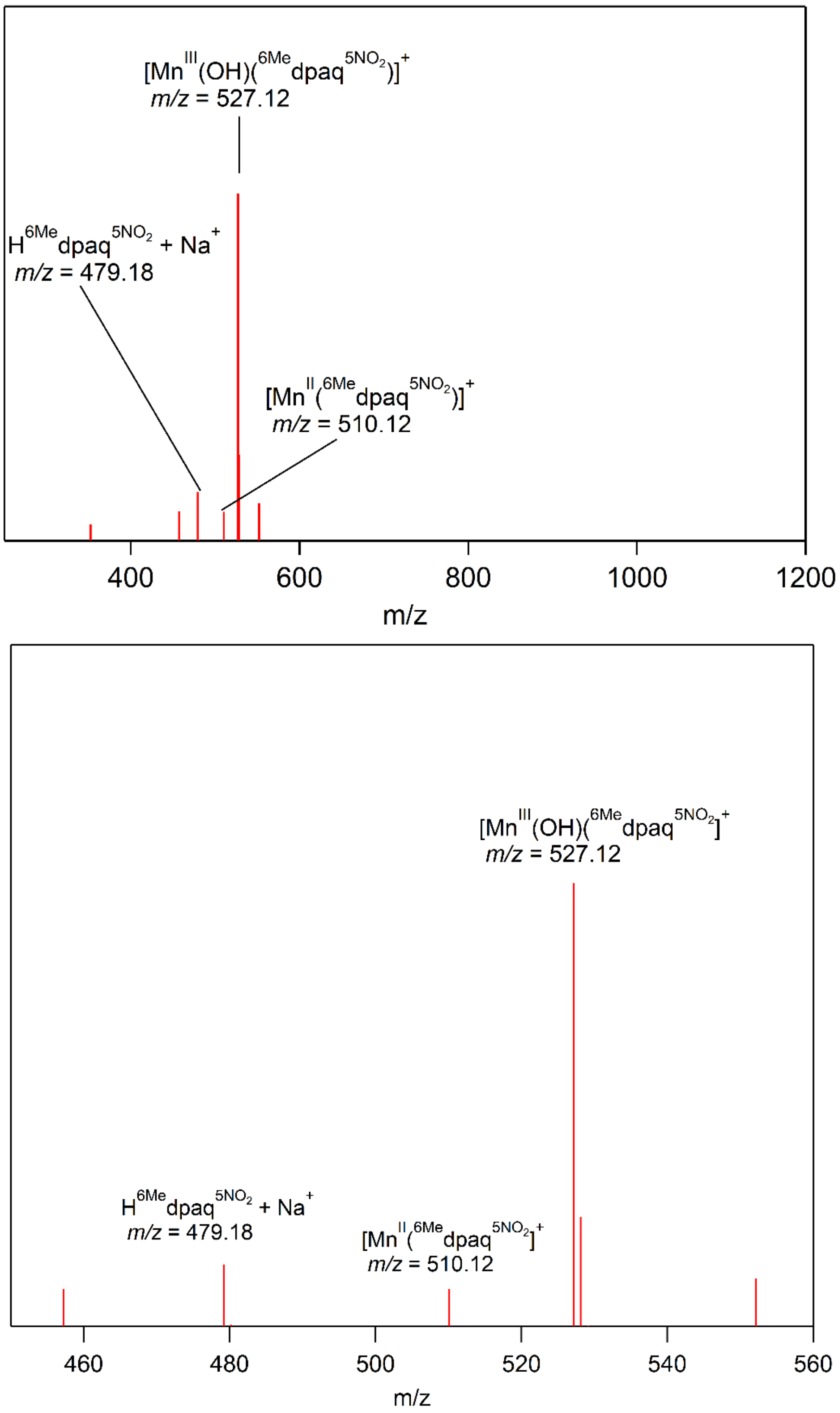

Figure S11. ESI-MS spectrum of **2** in CH_3CN. Both the full spectral region (top) and the region of interest (bottom) are shown.

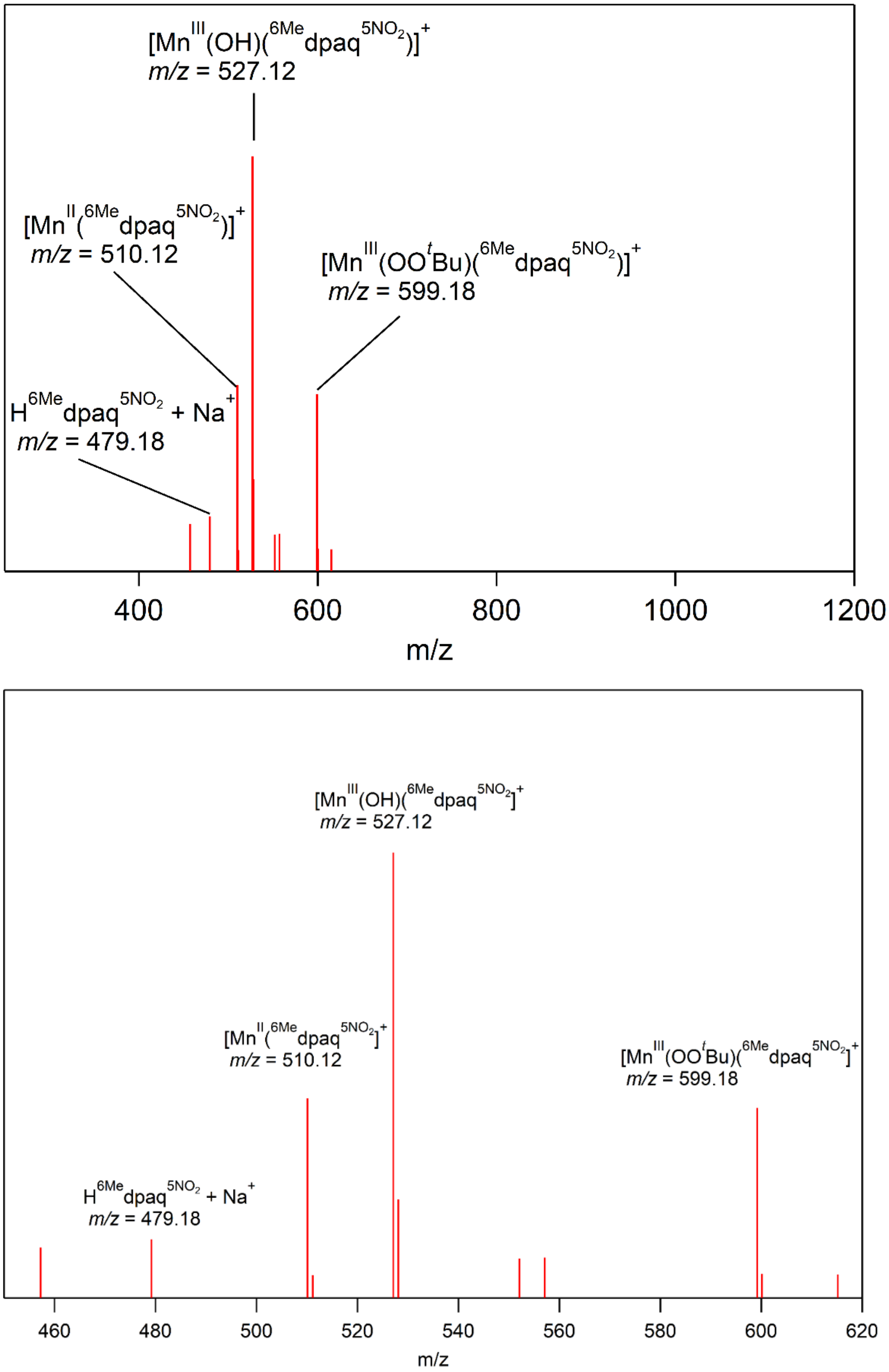

Figure S12. ESI-MS spectrum of **3a** in CH_3CN. Both the full spectral region (top) and the region of interest (bottom) are shown.

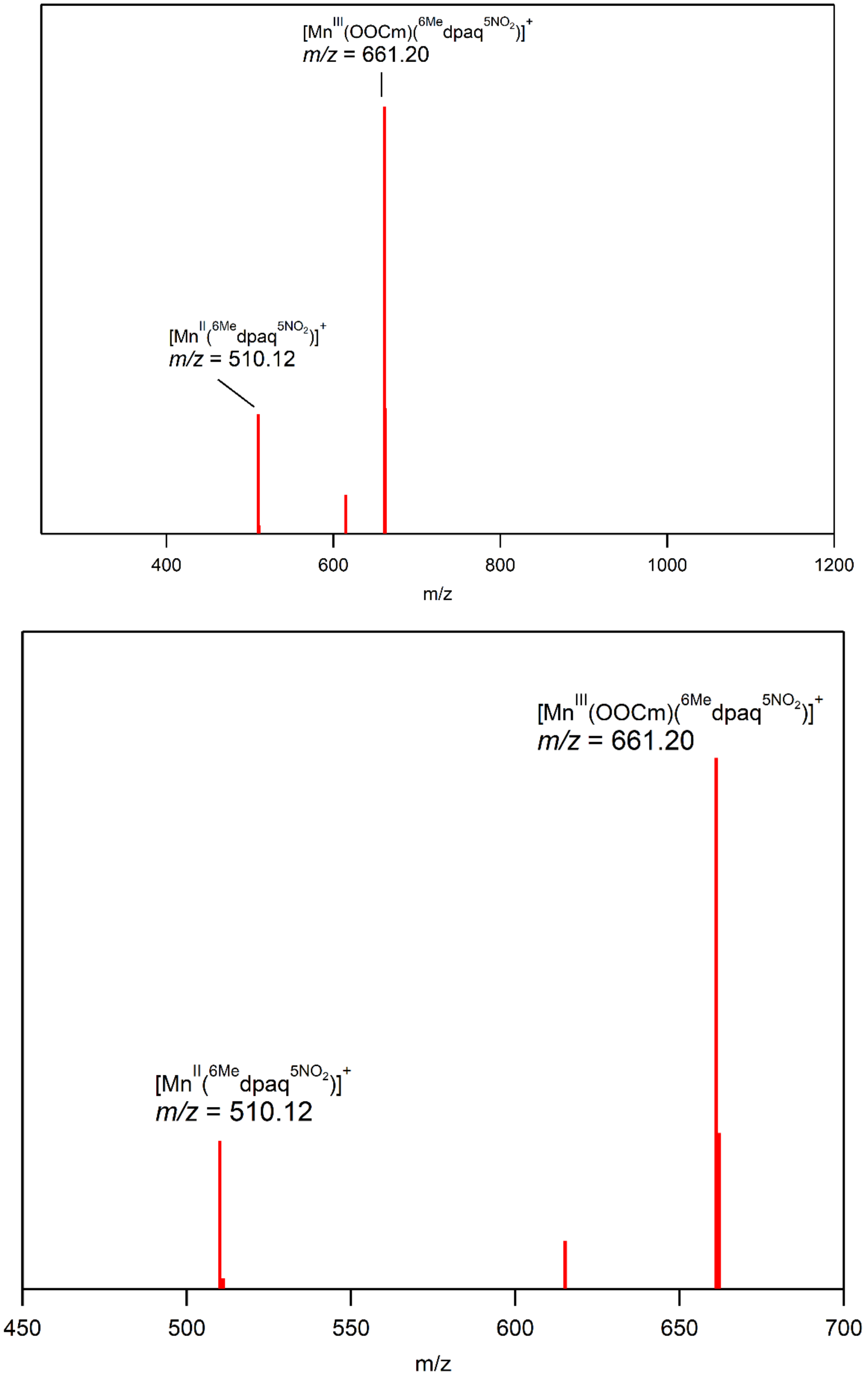

Figure S13. ESI-MS spectrum of **3b** in CH_3CN. Both the full spectral region (top) and the region of interest (bottom) are shown.

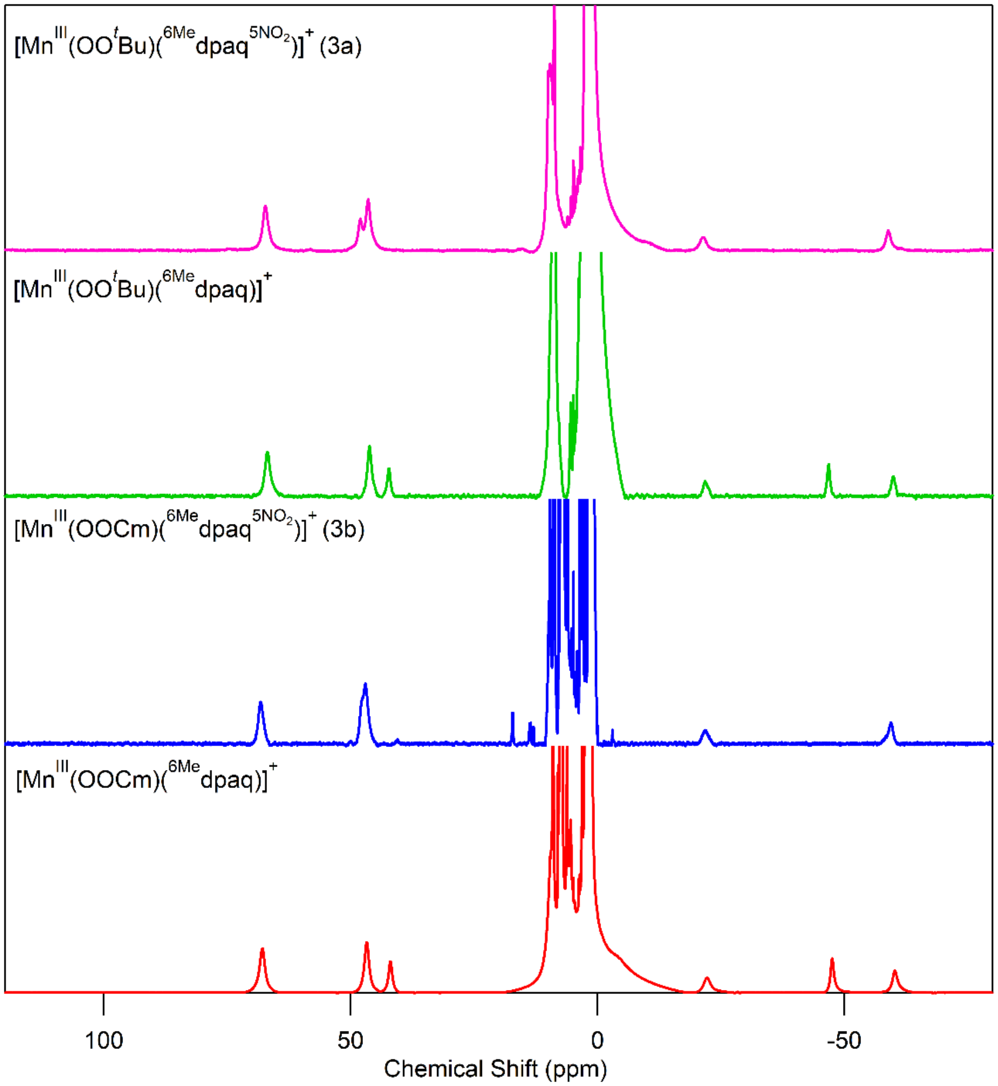

Figure S14. ^{1}H NMR spectra of **3a, 3b**, [Mn^{III}(OO^{t}Bu)(^{6Me}dpaq)]$^{+}$, and [Mn^{III}(OOCm)(^{6Me}dpaq)]$^{+}$ in CD_3CN at 298 K.

Table S1. ^{1}H NMR chemical shifts (ppm) for $[Mn^{III}(OH)(^{6Me}dpaq)]^+$, $[Mn^{III}(OO^tBu)(^{6Me}dpaq)]^+$, and $[Mn^{III}(OOCm)(^{6Me}dpaq)]^+$. Complexes in CD_3CN at 298 K.

$[Mn^{III}(OH)(^{6Me}dpaq)]^+$	$[Mn^{III}(OO^tBu)(^{6Me}dpaq)]^{+\,a}$	$[Mn^{III}(OOCm)(^{6Me}dpaq)]^{+\,a}$
66.0	66.8	67.8
51.4	46.1	46.7
44.8	42.2	41.8
8.9	9.2	8.9
		7.38
		7.26
		6.05
5.5		5.3
-9.6		
-19.3	-22.0	-22.3
-45.0	-46.9	-47.6
-61.6	-60.0	-60.3

[a] Data for these complexes from reference 48.

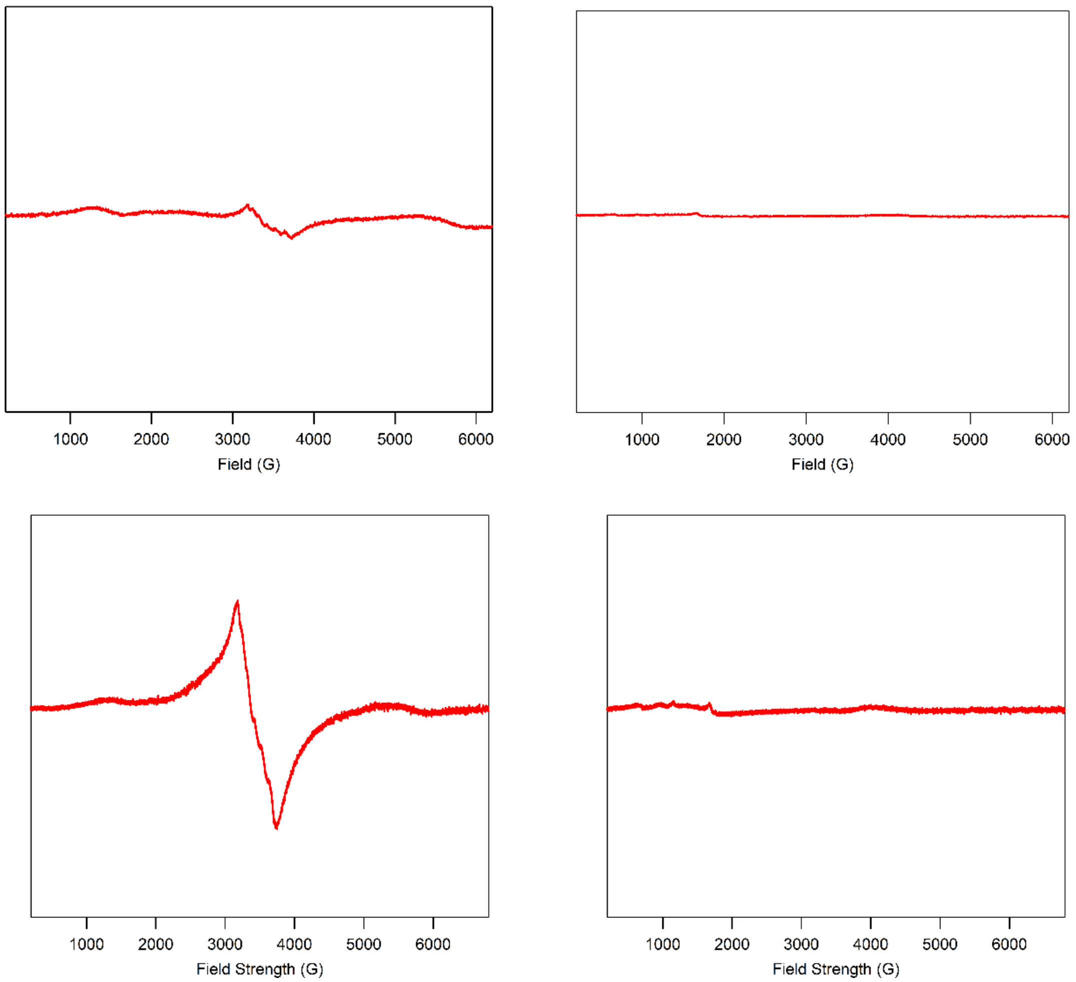

Figure S15. Top: X-band EPR spectra of frozen 3mM 1:1 acetonitrile:toluene solution of **3a** at 10 K in perpendicular-mode (left) and parallel-mode (right). Bottom: X-band EPR spectra of frozen 3 mM 1:1 acetonitrile:toluene solution of **3b** at 10 K in perpendicular-mode (left) and parallel-mode (right).

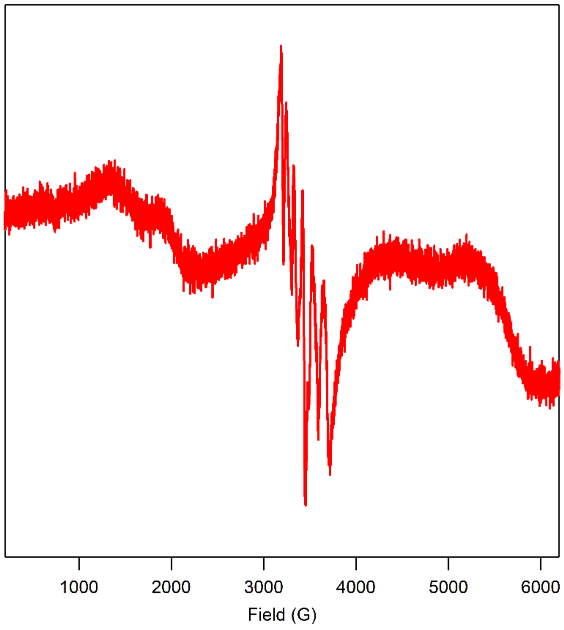

Figure S16. Perpendicular-mode, X-band EPR spectra of frozen 3mM solution of **3a** in acetonitrile at 8 K.

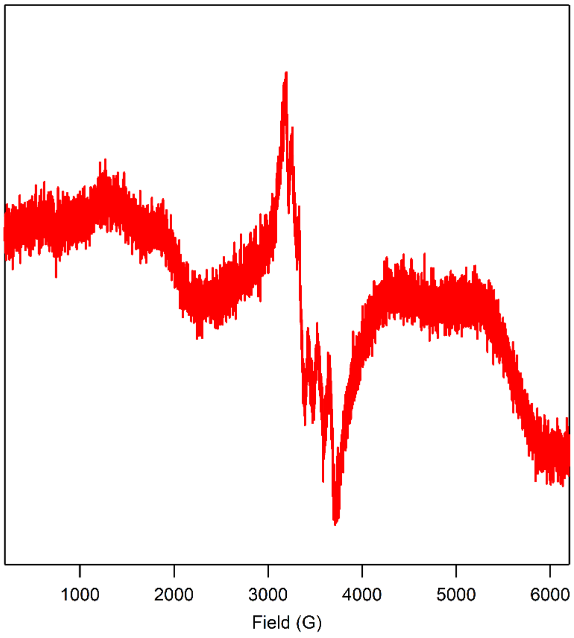

Figure S17. Perpendicular-mode, X-band EPR spectra of frozen 3 mM solution of **3b** in acetonitrile at 8 K.

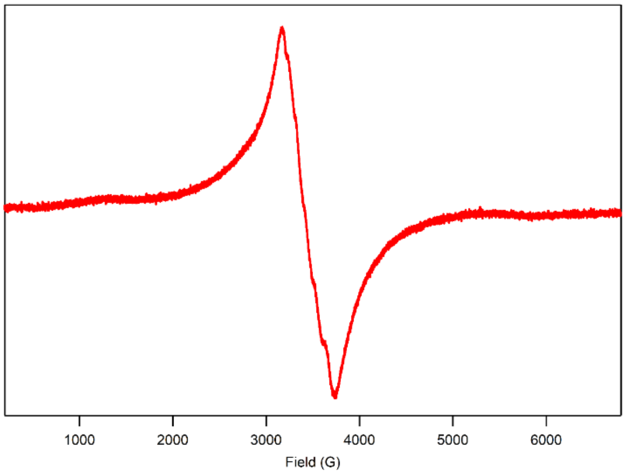

Figure S18. Perpendicular-mode, X-Band EPR spectrum at 7 K of the final products of thermal decay of a 3 mM solution of **3b** with a signal at g = 2.01.

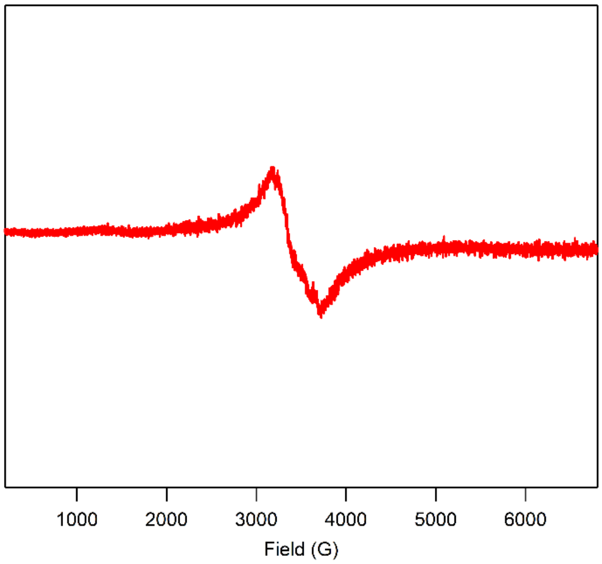

Figure S19. Perpendicular-mode, X-Band EPR spectrum at 8 K of the final products of thermal decay of **3a**.

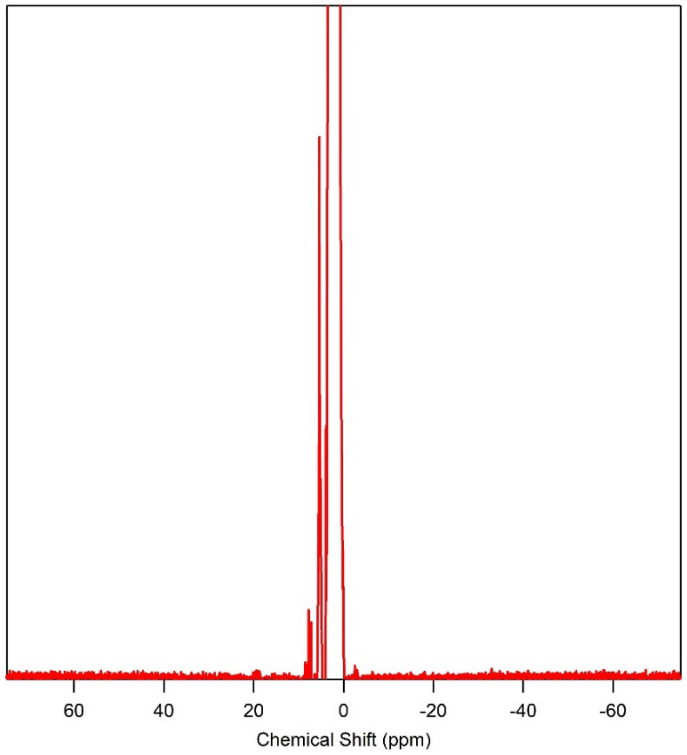

Figure S20. ¹H NMR spectra of the product of the reaction between **2** and 25 eq. TEMPOH in CD_3CN.

Reaction of 2 with triphenylphosphine

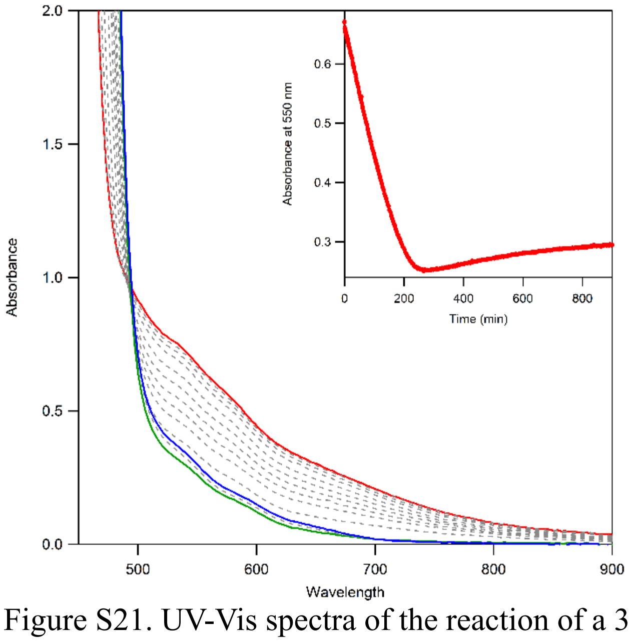

Figure S21. UV-Vis spectra of the reaction of a 3 mM solution of **2** with 10 equiv. triphenylphosphine at 308 K in atmosphere. Absorbance at 550 nm over time is shown in the inset. The initial trace is shown in red, minimum in green, and final in blue.

PPh_3 Oxidation by 2. The addition of 10 equiv. PPh_3 to a solution of **2** (3.0 mM in CH_3CN) at 308 K resulted in the slow loss of intensity of the feature at 550 nm over the course of *ca.* 4 hours, resulting in a spectrum consistent with a mixture of **2** and Mn^{II} products. After *ca.* 4 hours, the intensity of the 550 nm feature slowly increases. When performed anaerobically, the same loss of intensity happens but approximately 2 times slower than under atmosphere. In addition, in anaerobic experiments, we did not observe a growth in intensity at 550 nm; however, these trials were stopped after *ca.* 15 hours and the loss of intensity at 550 nm was still not complete at that time. For reactions performed under atmosphere, we analyzed the products by ESI-MS. These experiments showed the presence of peaks at both *m/z* of 262.09 and 278.09, consistent with PPh_3 and PPh_3O, respectively. The reaction product solution of the anerobic reaction was also analyzed using ESI-MS and showed only the presence of a peak at *m/z* 262.09, consistent with PPh_3 alone.

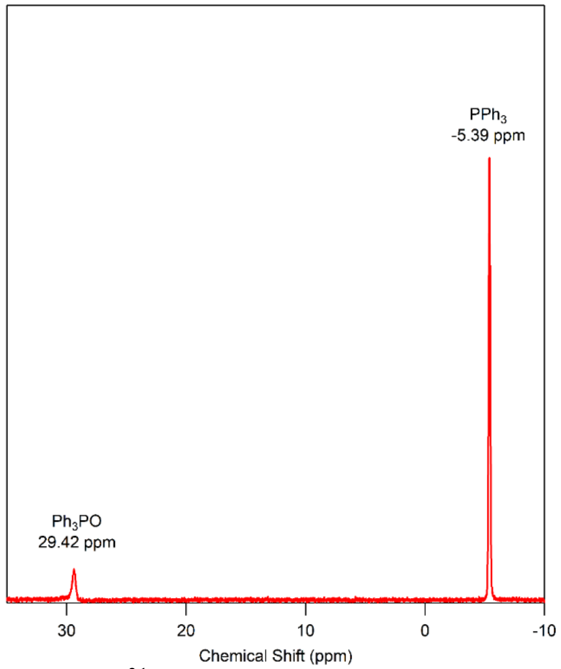

Figure S22. ^{31}P NMR of the organic products of the reaction between **3a** and PPh_3 in CD_3CN.

Single crystal X-ray diffraction analysis

Instrumentation: Single crystal X-ray diffraction analyses were carried out on a Bruker D8 Venture equipped with dual sealed tube MoKα (λ = 0.71073 Å) anodes operating at 50.0 kV and 1.40 mA (MoKα) and Helios multilayer X-ray optics.

Data collection and refinements: The single crystals were mounted on MicroMesh (MiTeGen) cryoloops with immersion oil at 100 K under a nitrogen cryostream. A total of 105685 and 36433 for **1** and **2**, respectively, using MoKα. Data were collected using 1° ω and ϕ-scans with 20-30 second exposures per frame using a PHOTON III C14 detector. Preliminary lattice constants and the intensities were integrated using the Bruker APEX4 program. The structures were determined by intrinsic phasing (SHELXT 2014/5)[1] and refined by full-matrix least-squares refinement on F2 (SHELXL-2017/1)[2] using the Olex2[3] software package using least-squares minimization. The final structural model incorporated anisotropic and isotropic atomic displacement parameters for non-hydrogen and hydrogen atoms, respectively. The hydrogen atoms attached to carbon atoms were eventually placed in calculated position and refine in the riding model sp^2- or sp^3-hybridized positions with C−H bond lengths of 0.95 - 0.99 Å. Relevant crystallographic and structure refinement data for labetalol hydrochloride is given in **Table S2**.

Table S2 Crystal data and structure refinement for 1 and 2

	**1**	**2**
Identification code	SAB-1J-146	SAB-1J-145
Empirical formula	$C_{26}H_{23}F_3MnN_6O_6S$	$C_{26}H_{24}F_3MnN_6O_7S$
Formula weight	659.509	676.51
Temperature/K	100	100
Crystal system	triclinic	monoclinic
Space group	P-1	$P2_1/n$
a/Å	9.2724(5)	8.824(2)
b/Å	9.3267(5)	24.170(5)
c/Å	17.5457(10)	13.339(3)
α/°	92.824(2)	90
β/°	103.391(2)	102.634(7)
γ/°	111.925(2)	90
Volume/Å^3	1353.77(13)	2775.9(11)
Z	2	4
ρ_{calc}g/cm^3	1.618	1.619
μ/mm^{-1}	0.640	0.629
F(000)	675.5	1384.0
Crystal size/mm^3	0.2 × 0.1 × 0.05	0.1 × 0.05 × 0.05
Radiation	Mo Kα (λ = 0.71073)	MoKα (λ = 0.71073)
2Θ range for data collection/°	5.32 to 72.64	4.6 to 49.422
Index ranges	-15 ≤ h ≤ 15, -15 ≤ k ≤ 15, -29 ≤ l ≤ 29	-10 ≤ h ≤ 10, -28 ≤ k ≤ 28, -15 ≤ l ≤ 15
Reflections collected	105685	36433
Independent reflections	12874 [Rint = 0.0301, Rsigma = 0.0175]	4723 [R_{int} = 0.1354, R_{sigma} = 0.1010]
Data/restraints/parameters	12874/0/408	4723/0/400
Goodness-of-fit on F^2	1.057	1.125
Final R indexes [I>=2σ (I)]	R1 = 0.0309, wR2 = 0.0856	R_1 = 0.0682, wR_2 = 0.1531
Final R indexes [all data]	R1 = 0.0324, wR2 = 0.0867	R_1 = 0.0977, wR_2 = 0.1650
Largest diff. peak/hole / e Å^{-3}	0.66/-0.75	0.61/-0.44
CCDC	2339541	2339539

Powder X-ray diffraction (PXRD) analysis

Instrumentation and measurement method: The XRD spectrums were collected by a fine-focus Mo-sealed tube running at 50 kV and 1.1 mA (Cu Kα = 1.54178 Å) on a Bruker D8 Venture Diffractometer equipped with Helios multilayer optics, an Burker Photon III C14 detector. Sample preparation included mixing powder with Paratone N oil and placing it in a <0.5 mm nylon loop mounted on a goniometer head. A Bruker APEX4 software package was used to collect and merge, 40 s. 360° φ-scans with the detector at 2θ = -30°, -45°, 30° and 45°using a sample-to-detector distance of 100 mm.

PXRD Spectra:

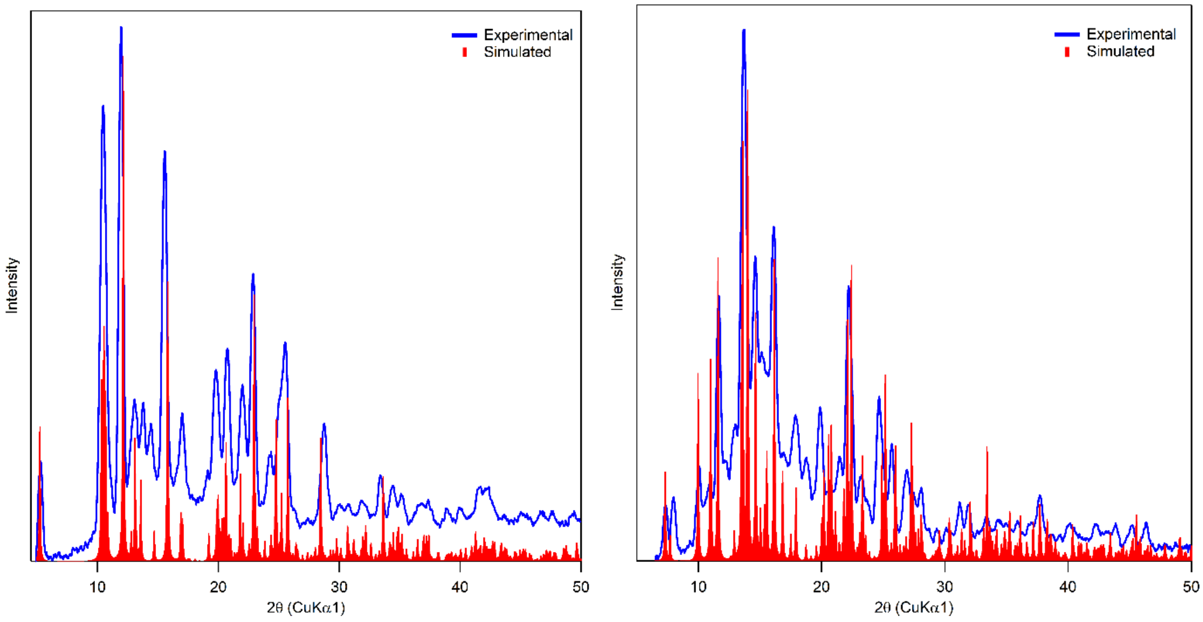

Figure S23. PXRD Spectra of **1** (left) and **2** (right), experimental is shown in blue while simulated is shown in red.

[Mn^{III}(OO^tBu)($^{6Me}dpaq^{5NO_2}$)]$^+$ (3a)

Mn	-0.08474677315861	-0.14857788643680	0.00481044839694
O	-5.92781183107275	1.65851981999330	-3.57996972650446
O	-4.80444697698100	2.39543988208267	-5.28277163001961
O	-0.68770434766363	0.77509794604490	2.42247477820771
O	1.50004667494647	0.13722511923036	-3.73174625783758
O	-0.25522705326730	-0.46705302168470	1.80182395785131
N	-4.87826012793191	1.86457404865627	-4.18453405215594
N	0.98784311849810	1.93631926242543	0.12036527991743
N	1.99327478315709	-0.66672023566677	-0.21121548153634
N	0.02697655206003	0.09097644176930	-1.93512505939380
N	-0.02246112078920	-2.49213685992706	-0.22346452610706
N	-2.02485756566192	0.36332759892088	-0.39264962235886
C	-0.20597348680620	0.83532802032914	3.79743052872554
C	-1.02194159290311	3.33698336039156	0.12436357134238
C	-2.33208986945809	-2.78243994670626	-0.97776130891274
C	0.45826348662525	3.16388916011995	-0.04869970719244
C	1.27712316249754	4.25917972787754	-0.36615937963166
C	2.64947440278397	4.08140645082954	-0.51379556915076
C	3.18796396536950	2.80926945067860	-0.30570024389272
C	2.31949630929378	1.77136403880067	0.02151890895162
C	2.82072992013620	0.40261473189509	0.39071666006958
C	-0.99132926587116	-3.34643131850048	-0.61168026522566
C	-0.74568398584784	-4.72517326642497	-0.67589919442051
C	0.51452728742269	-5.22144300899229	-0.34943560756150
C	1.51267354156022	-4.32378959799363	0.03209312254727
C	1.19570019038369	-2.96739200799750	0.08675152319795
C	2.20534164834820	-1.93802600660647	0.52050835737189
C	2.25807041151706	-0.80677755669456	-1.67456454207765
C	1.23016058246592	-0.11214263115069	-2.56749693396995
C	-2.24707943481267	0.68090017170463	-1.70616642248893
C	-1.10733606857602	0.56313693500246	-2.57046464356941
C	-1.25864959353211	0.90848714633387	-3.91418940602750
C	-2.51095375749615	1.34334418161750	-4.38198474582036
C	-3.62023300660779	1.42890095068031	-3.55709860544046
C	-3.53005144662456	1.10002256033462	-2.16777398328891
C	-4.56314752121082	1.17377902629737	-1.19032589131999
C	-4.29801904643904	0.83665401638630	0.12486814856377
C	-3.00631665795660	0.42905515491926	0.49727755616364
H	-1.38025826282066	2.71646481143871	0.95483917317670
H	-1.26682377429066	4.38871034698643	0.32157170082927
H	-1.55713082528302	3.03818011904610	-0.79009051321678
H	-2.22846517965100	-2.02651091971144	-1.76919081125295
H	-3.00413150680762	-3.57217614322820	-1.33538298953040
H	-2.79686086553419	-2.29782944625386	-0.10726712035884
H	0.82471815407175	5.24328730155771	-0.49642082632061

H	3.29429009542660	4.92204378608947	-0.77666184971549
H	4.26037662419941	2.62572071717986	-0.38654120980505
H	2.74090403979736	0.29327742436084	1.48119570333834
H	3.87840266162012	0.27723591594768	0.10883664511195
H	-1.54520707118562	-5.39668553742378	-0.99130067914474
H	0.72057926225505	-6.29215226420140	-0.40304227113962
H	2.51893140781072	-4.66461441128829	0.28064290574542
H	3.23247492690470	-2.31107069938961	0.37457744115425
H	2.06530559998176	-1.71972107338220	1.59013572200589
H	3.26361328803135	-0.44191300916272	-1.92654256458671
H	2.21848456415843	-1.87440635039659	-1.93657044567749
H	-0.41062924258234	0.83586475381431	-4.58992266545437
H	-2.63380174345271	1.61640386051492	-5.43001108832780
H	-5.55725405784648	1.49225625987296	-1.49397719294822
H	-5.08307037638166	0.88867748158586	0.87993434268652
H	-2.74290645801935	0.17179446283828	1.52308319563910
C	1.32159207959623	0.83127832160726	3.82334850530762
H	1.70922341643922	-0.13391241246366	3.46603063104724
H	1.68394593162770	0.97971247659014	4.85131511195707
H	1.71915521488583	1.64202693314699	3.19452833404742
C	-0.79433284858153	-0.32495134267412	4.59830030240891
H	-0.45439533737682	-1.28695589834860	4.18970602977627
H	-1.89368754506771	-0.29735879934074	4.56508166796854
H	-0.47709602051507	-0.26093019276620	5.65004913765127
C	-0.76559190955144	2.18471804335376	4.25111975635528
H	-0.34822725018139	3.00375005477942	3.64668754809720
H	-0.50098746463049	2.35890586211874	5.30441146151685
H	-1.86179503344364	2.19964770866258	4.16318687625567

$[Mn^{III}(OOCm)(^{6Me}dpaq^{5NO_2})]^+$ (3b)

Mn	0.11518773993508	-0.52408356640229	0.01641952515964
O	-0.30266903281375	-0.91091334630846	1.75292864611730
O	1.98127530175803	-0.34703796777559	-3.60124417539068
O	-0.41925835418791	0.27356218282580	2.58206509112280
O	-3.79071382121459	2.80009858468697	-5.50916988643114
O	-5.00426894686127	2.66162607797383	-3.71943547088315
N	-1.70923267377798	0.23743045662132	-0.49644282402950
N	0.09279463401384	-2.79875925843493	-0.09263882829243
N	0.39799932135189	-0.23627293974865	-1.89978931448128
N	2.18036452214336	-1.14435889533492	-0.07766073226778
N	1.24871046061663	1.52200264690148	0.25991655062450
N	-3.96772117210570	2.45986571107470	-4.34828940907774
C	-0.43245653081738	1.14845459603254	4.73979020389815
C	-1.39110310659799	-1.13758619049900	4.31228213805316
C	-2.74472841193974	0.36961509883319	0.32169987156358

C	-3.93534561786364	0.97410995824012	-0.11148940142001
C	-4.03603539457942	1.45546851901179	-1.40532888407291
C	-2.93950223504672	1.32467125546703	-2.30549501335849
C	-2.86073016261450	1.76319767218441	-3.66690666862349
C	-1.71460598350705	1.54781563632324	-4.41556154630694
C	-0.58551417426023	0.89250910229875	-3.89736820527698
C	-0.59778146865572	0.44319207757296	-2.57652924998974
C	-1.77455625775212	0.67961757195552	-1.79013811751418
C	1.60409891644705	-0.58683628412860	-2.46472129558402
C	2.45130330818018	-1.43197254971403	-1.51598239515686
C	2.30517554212428	-2.34626574703863	0.77759825469797
C	1.19779337982135	-3.32090427612941	0.46618971257469
C	1.31192286573471	-4.67990501685062	0.75420880301792
C	0.23591251302110	-5.51407590642650	0.44971390740369
C	-0.89703040448607	-4.96809398388444	-0.15365924187527
C	-0.94506559789318	-3.59462671781037	-0.42003190120866
C	3.05106473777427	-0.04966113939823	0.41203343740108
C	2.54102662725003	1.28843466156031	-0.04912387637760
C	3.34470140374265	2.22893729067411	-0.68260307868857
C	2.78815108250365	3.47663022070792	-0.98629435837691
C	1.47872588338004	3.73932958404868	-0.60017477864477
C	0.72329332222593	2.74030537966420	0.03830142006032
C	-2.13570885664461	-2.95346172470410	-1.07117626898583
C	-0.67252248475550	3.01648979673820	0.52164215299882
C	-0.26569399336760	-0.16413496907628	3.96728158755865
H	-1.33276493530848	-1.44592156222387	5.36618869456812
H	-2.35835933090088	-0.63772188206412	4.15523440766636
H	-1.35119626877698	-2.03191768048995	3.67699585470207
H	-0.28299200705426	0.96374294829545	5.81332648625059
H	0.29678477958093	1.90152519135799	4.41240357012645
H	-1.44436014582370	1.55181324586093	4.58668796343616
H	-2.60454712099385	-0.00185248374691	1.33705063040152
H	-4.77263473962986	1.07060764227942	0.58056131786900
H	-4.94584027254979	1.93909305113593	-1.75029158936613
H	-1.70875937091555	1.90617005409651	-5.44440245628846
H	0.29240763603989	0.72918688527671	-4.51670966882486
H	2.19443097649234	-2.48225087260678	-1.71636944797069
H	3.51632172542917	-1.28987986409618	-1.74892162687603
H	2.21612096133248	-2.01767650276515	1.82429237950647
H	3.29030606300113	-2.82619048494882	0.64946131316640
H	2.22566777899301	-5.07204447471093	1.20383822134571
H	0.28619297174738	-6.58281231254571	0.66747528854720
H	-1.74338354037518	-5.59907774860957	-0.42877923925042
H	4.09253511249676	-0.21461471346253	0.09426344933320
H	3.01217436743922	-0.07600560301981	1.50937140571847
H	4.37907421159333	1.99200984125928	-0.93490691406453

H	3.37876157683096	4.23713182083781	-1.50060589635541
H	1.02823859671098	4.71584535383695	-0.78499930254279
H	-2.72810995415328	-2.40123730766508	-0.32505864287402
H	-2.78590749467300	-3.70702581530020	-1.53398305027154
H	-1.81428023507585	-2.23771644389040	-1.84048311134456
H	-1.39640414280472	2.97667088313212	-0.30788986796373
H	-0.72771073855292	4.02800046658985	0.95005262560997
H	-0.95766629317296	2.28611231340111	1.28694332749179
C	1.13737509603888	-0.74857666462569	4.13583889550824
C	1.35723580678661	-2.08923033121673	4.48339302692723
H	0.51225317148105	-2.75499929841527	4.65803103970648
C	2.65668341404003	-2.59212686002252	4.62463339978409
H	2.80358472756423	-3.63594597867560	4.91105928756007
C	3.76048105684743	-1.75964366100853	4.42598417638426
H	4.77310324412170	-2.14712109085298	4.55807375900820
C	3.55480506347297	-0.41970841985662	4.07688024045810
H	4.40981042020881	0.24458801319302	3.93116770302474
C	2.25654348579219	0.07577110778291	3.92771494038087
H	2.10915746643836	1.12031563675203	3.64483102957392

References

1. Sheldrick, G.M. SHELXT - integrated space-group and crystal-structure determination. *Acta Crystallogr A Found Adv* **2015**, *71*, 3-8, doi:10.1107/S2053273314026370.
2. Sheldrick, G.M. Crystal structure refinement with SHELXL. *Acta Crystallogr C Struct Chem* **2015**, *71*, 3-8, doi:10.1107/S2053229614024218.
